# A topological nonlinear parametric amplifier

**DOI:** 10.1038/s41467-022-34979-y

**Published:** 2022-11-24

**Authors:** Byoung-Uk Sohn, Yue-Xin Huang, Ju Won Choi, George F. R. Chen, Doris K. T. Ng, Shengyuan A. Yang, Dawn T. H. Tan

**Affiliations:** 1 Photonics Devices and System Group, Singapore University of Technology and Design, Singapore, 487372 Singapore; 2 Research Laboratory for Quantum Materials, Singapore University of Technology and Design, Singapore, 487372 Singapore; 3 Institute of Microelectronics, A*STAR, 2 Fusionopolis Way, #08-02, Innovis Tower, Singapore, 138634 Singapore

**Keywords:** Optical physics, Nonlinear optics

## Abstract

Topological boundary states are well localized eigenstates at the boundary between two different bulk topologies. As long as bulk topology is preserved, the topological boundary mode will endure. Here, we report topological nonlinear parametric amplification of light in a dimerized coupled waveguide system based on the Su-Schrieffer-Heeger model with a domain wall. The good linear transmission properties of the topological waveguide arising from the strong localization of light to the topological boundary is demonstrated through successful high-speed transmission of 30 Gb/s non-return-to-zero and 56 Gb/s pulse amplitude 4-level data. The strong localization of a co-propagating pump and probe to the boundary waveguide is harnessed for efficient, low power optical parametric amplification and wavelength conversion. A nonlinear tuning mechanism is shown to induce chiral symmetry breaking in the topological waveguide, demonstrating a pathway in which Kerr nonlinearities may be applied to tune the topological boundary mode and control the transition to bulk states.

## Abstract (web summary)

Here the authors report the development of a topological nonlinear parametric amplification in a dimerized, Su-Schrieffer-Heeger waveguide. Kerr-induced chiral symmetry breaking is demonstrated, showcasing how nonlinearities may control transitions of topological modes to bulk states.

## Introduction

Advancements in topological photonics are taking place at breakneck speeds. One of the earliest observations of topological behavior was the Hall conductance in a 2D system in the presence of a magnetic field which breaks time-reversal symmetry^1^. In a similar vein as the aforementioned anomalous Hall conductance in periodic systems, topological solitons in polyacetylene, now broadly referred to as the Su–Schrieffer–Heeger (SSH) system^2,3^ broke new ground in condensed matter physics. Thereafter, the field of topological insulators experienced widespread proliferation when the role of various symmetries in topological states was introduced^4,5^. In 2008, the topological bridge between condensed matter physics and photonics was firmly cemented, when the photonic equivalent of the Quantum-Hall-effect was theorized, predicting the conditions required for unidirectional propagation of light in a photonic crystal^6^. Shortly after, these theoretical predictions were reduced to practice experimentally by Wang et al.^7,8^. These milestone works were the first of many tantalizing topological photonic designs to come, including those analogous to the quantum Hall^9^, quantum spin Hall^10–13^, quantum valley Hall^14–16^, and Weyl singular point^17,18^ systems. Recent strides in topological photonics show great promise for a variety of applications, including topological quantum light generation^19–21^, topological lasers^22–24^, and advanced photonic routing^25–29^.

Importantly, the fusion of the fields of silicon photonics and topological photonics could be significantly transformative for two reasons: (i) Such a step would open up a plethora of potential applications prolific in silicon photonics^30–34^ in which topological photonics could find much-needed commercial relevance. (ii) The implementation of topological photonic devices and systems on silicon-based platforms is especially pertinent for their accessibility to CMOS manufacturing and their ease of integration with application-specific integration circuits. The study of optical nonlinearities is a core and integral part of contemporary integrated silicon photonics, providing an irreplaceable avenue for ultrafast control of light and light generation in the absence of a direct bandgap.

Fortuitously, nonlinearities in topological photonic systems are currently a domain of active research and could be a key area in which to harmonize advantageously with silicon photonics. Recent studies of nonlinearities in topological photonics have unveiled new frameworks for soliton formation^35–38^, non-Hermitian topological systems, and their manipulation^39–42^.

In this manuscript, we demonstrate parametric amplification and wavelength conversion in a topological photonic waveguide based on the SSH model with a domain wall, implemented on CMOS-compatible ultra-silicon-rich nitride (USRN). The linear transmission properties of the topological waveguide are characterized using high-speed testing and shown to support the high-speed transmission of 30 Gb/s non-return-to-zero (NRZ) and 56 Gb/s pulse amplitude modulation 4-level (PAM4) data. Using the designed topological waveguides, we report 12.8 dB parametric gain and wavelength conversion spanning 100 nm. Owing to a sublattice/chiral symmetry of the waveguide design, the topological mode has its amplitude suppressed in the odd waveguides within the array. We demonstrate that the Kerr-induced on-site perturbation in the local refractive index breaks the chiral symmetry, resulting in an increased amplitude in the odd waveguides and a spatial broadening of the topological mode. Notably, this effect is observed at low peak powers of tens of watts, making this topological control mechanism easily accessible. Our experimental results and theoretical analyses showcase a pathway in which a topological boundary mode may be harnessed for nonlinear parametric light generation and likewise be controlled through nonlinear optical effects at low powers.

### Theory of USRN topological waveguides based on the SSH model with a domain wall

**Fig. 1:**
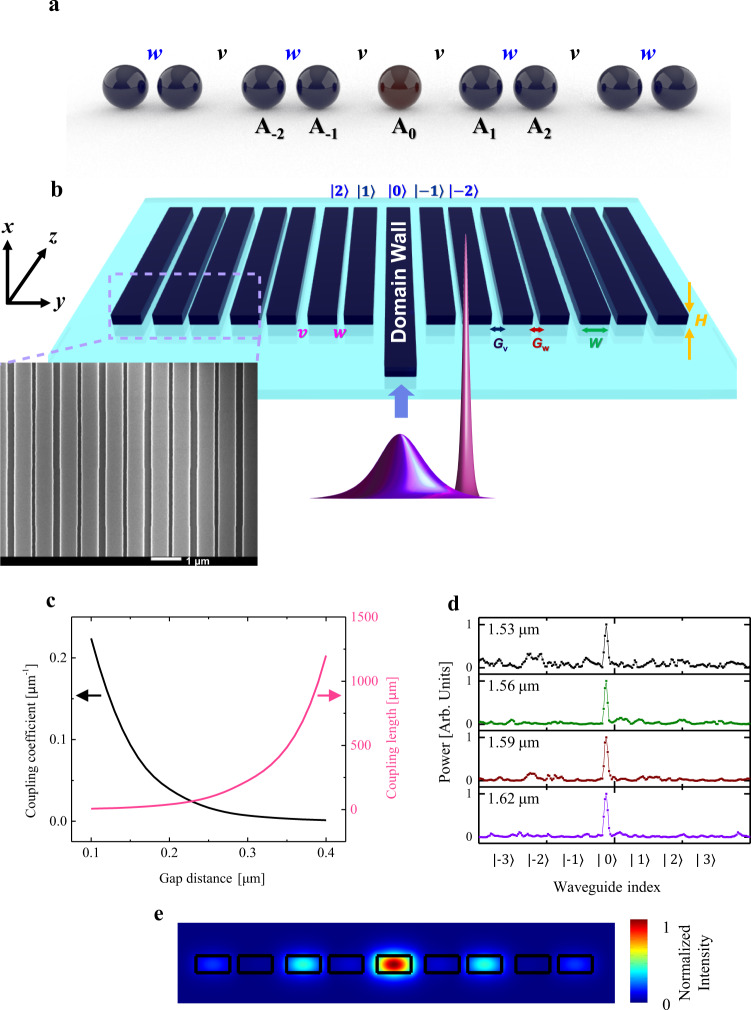
The SSH model, topological waveguide schematic, and calculated coupling coefficients between two waveguides. **a** Diagram of the dimer chain making up the SSH model with a domain wall. **b** Device schematic and scanning electron micrograph of the USRN topological waveguide. **c** Coupling coefficient and coupling length for two coupled waveguides as a function of gap distance for a size of 0.6 µm × 0.3 µm waveguide fabricated with a USRN core and SiO_2_ cladding. **d** Measured output powers versus waveguide index positions at diverse wavelengths from 1.53 to 1.62 µm. The output power is observed to be well localized at the boundary waveguide indexed to be |0〉. **e** Topological mode profile numerically calculated using the finite-element method. It is observed that the mode is well-localized to the topological domain wall and as expected from the chiral symmetry, the amplitude at waveguides |±1〉 is suppressed.

Topological waveguides leverage the zero modes generated at the boundary between two topological domains of dimerized lattices. It is well known that in the 1D SSH model, each unit cell contains two sites, and the topology is determined by the relative strengths of the intracell coupling *v* and the intercell coupling *w*: the topology is non-trivial (trivial) when *v* < *w* (*v* > *w*) (see Supplementary Note 9 for details). The boundary between topologically trivial and nontrivial regions represents a topological domain wall and must host a topological mode that peaks at the boundary. One representative domain wall configuration is shown in Fig. 1a, where the dimerization pattern is swapped between the left and right domains so that a topological mode must appear at the center. In the following, we shall see that the physics of our photonic waveguide system can be mapped to this configuration of a domain wall in the SSH model. Full details of the theory may be found in Supplementary Note 1.

In a waveguide, the propagation dynamics of the electric field are described by the nonlinear Schrödinger equation as follows^43^:

1 $$i\frac{\partial {{{{{\bf{E}}}}}}}{\partial z}+i\alpha {{{{{\bf{E}}}}}}+\frac{1}{2{{{{{{\rm{\beta }}}}}}}_{0}}{{{{{{\boldsymbol{\nabla }}}}}}}_{\perp }^{2}{{{{{\bf{E}}}}}}+\frac{{k}_{0}^{2}{n}_{0}^{2}-{\beta }_{0}^{2}+2\,{k}_{0}^{2}{n}_{0}{{{{{{\bf{n}}}}}}}_{2}{|{{{{{\bf{E}}}}}}|}^{2}}{2{\beta }_{0}}{{{{{\bf{E}}}}}}=0$$

Here, *n*_0_(*x, y*) is a spatially varying refractive index distribution function and corresponds to a periodic potential in the *y*-direction (see Fig. 1a for *x*- and *y*-axes orientations). ${{{{{{\bf{n}}}}}}}_{2}=\frac{3}{4}{{{{{{\rm{\epsilon }}}}}}}_{0}{{{{{{\boldsymbol{\chi }}}}}}}^{(3)}$ is the nonlinear Kerr effect, *z* is the propagation direction, *α* is the loss coefficient and *β*_0_ is the wave number corresponding to a fast-varying phase. We choose ${\beta }_{0}={n}_{{{{{{\rm{eff}}}}}}}\,{k}_{0}$ of a boundary mode to make it a zero mode, where ${k}_{0}=\frac{2{{{{{\rm{\pi }}}}}}}{\lambda }$ and **E** is the slowly varying amplitude. We further assume that ${{{{{{\bf{n}}}}}}}_{2}{|{{{{{\bf{E}}}}}}|}^{2} \,\ll\, {n}_{0}$. Considering the peak power used in our experiments and the nonlinear refractive index of our material, the refractive index variation from the Kerr effect, $\Delta n={{{{{{\bf{n}}}}}}}_{2}{|{{{{{\bf{E}}}}}}|}^{2} \, < \, 0.01$. Boundary problems for eigenvalues and eigenstates and propagation dynamics can be solved using the aforementioned nonlinear Schrödinger equation (see Supplementary Notes 10, 11 for details). The slowly varying envelope approximation is justified in Supplementary Note 15.

The nonlinear Schrödinger equation is used to analyze propagation dynamics involving a single propagating field, which we discuss later in the manuscript. Where parametric processes are concerned with involving a pump and signal, coupled equations are required for their theoretical treatment. For a description of the parametric process, the field, *E* is replaced by four fields ${{{{{\bf{E}}}}}}={{{{{{\bf{E}}}}}}}_{1}{{\rm {e}}}^{{\rm {i}}\,{\beta }_{1}z}+{{{{{{\bf{E}}}}}}}_{2}{{\rm {e}}}^{{\rm {i}}\,{\beta }_{2}z}+{{{{{{\bf{E}}}}}}}_{s}{{\rm {e}}}^{{\rm {i}}\,{\beta }_{s}z}+{{{{{{\bf{E}}}}}}}_{i}{{\rm {e}}}^{{\rm {i}}\,{\beta }_{i}z}+{{{{{\rm{c}}}}}}.{{{{{\rm{c}}}}}}.$ For degenerate pump fields, **E**_1_ = **E**_2_ = **E**_*p* _corresponding to our experimental conditions, giving rise to three coupled equations:

2 $$i2{\beta }_{0p}\frac{\partial {{{{{\bf{E}}}}}}_{p}}{\partial z} 	+{{{{{{\boldsymbol{\nabla }}}}}}}_{\perp }^{2}{{{{{\bf{E}}}}}}_{p}+({k}_{0p}^{2}{n}_{0}^{2}-{\beta }_{0p}^{2}){{{{{\bf{E}}}}}}_{p}+2{k}_{0p}^{2}{n}_{0}{{{{{\bf{n}}}}}}_{2}\left({|{{{{{\bf{E}}}}}}_{p}|}^{2}+2{|{{{{{\bf{E}}}}}}_{s}|}^{2}+2{|{{{{{{\bf{E}}}}}}}_{i}|}^{2}\right){{{{{\bf{E}}}}}}_{p} \\ 	+4{k}_{0p}^{2}{n}_{0}{{{{{{\bf{n}}}}}}}_{2}{{{{{\bf{E}}}}}}_{p}^{*}{{{{{\bf{E}}}}}}_{s}{{{{{\bf{E}}}}}}_{i}{{\rm {e}}}^{{\rm {i}}\triangle \beta z}=0$$

3 $$i2{\beta }_{0s}\frac{\partial {{{{{{\bf{E}}}}}}}_{s}}{\partial z} 	+{{{{{{\boldsymbol{\nabla }}}}}}}_{\perp }^{2}{{{{{{\bf{E}}}}}}}_{s}+({k}_{0s}^{2}{n}_{0}^{2}-{\beta }_{0s}^{2}){{{{{{\bf{E}}}}}}}_{s}+2{k}_{0s}^{2}{n}_{0}{{{{{{\bf{n}}}}}}}_{2}\left({|{{{{{{\bf{E}}}}}}}_{s}|}^{2}+2{|{{{{{{\bf{E}}}}}}}_{p}|}^{2}+2{|{{{{{{\bf{E}}}}}}}_{i}|}^{2}\right){{{{{{\bf{E}}}}}}}_{s} \\ 	+4{k}_{0s}^{2}{n}_{0}{{{{{{\bf{n}}}}}}}_{2}{{{{{{\bf{E}}}}}}}_{s}^{*}{{{{{{\bf{E}}}}}}}_{p}{{{{{{\bf{E}}}}}}}_{i}{{\rm {e}}}^{-i\triangle \beta z}=0$$

4 $$i2{\beta }_{0i}\frac{\partial {{{{{{\bf{E}}}}}}}_{i}}{\partial z} 	+{{{{{{\boldsymbol{\nabla }}}}}}}_{\perp }^{2}{{{{{{\bf{E}}}}}}}_{i}+({k}_{0i}^{2}{{{{{{\rm{n}}}}}}}_{0}^{2}-{\beta }_{0i}^{2}){{{{{{\bf{E}}}}}}}_{i}+2{k}_{0i}^{2}{n}_{0}{{{{{{\bf{n}}}}}}}_{2}\left({\left|{{{{{{\bf{E}}}}}}}_{i}\right|}^{2}+2{\left|{{{{{{\bf{E}}}}}}}_{p}\right|}^{2}+2{\left|{{{{{{\bf{E}}}}}}}_{s}\right|}^{2}\right){{{{{{\bf{E}}}}}}}_{i} \\ 	+4{k}_{0i}^{2}{n}_{0}{{{{{{\bf{n}}}}}}}_{2}{{{{{{\bf{E}}}}}}}_{i}^{*}{{{{{{\bf{E}}}}}}}_{p}{{{{{{\bf{E}}}}}}}_{s}{{\rm {e}}}^{-i\triangle \beta z}=0$$

where $$\Delta \beta={\beta }_{0i}+{\beta }_{0s}-2{\beta }_{0p}$$.

The fourth term in each of the three coupled equations is composed of self-phase modulation and cross-phase modulation terms. The degree of tuning of the topological state for the signal is twice as strong because of the factor of 2 associated with $2{|{{{{{{\bf{E}}}}}}}_{p}|}^{2}$ in Eq. (3) (interaction between the signal and pump), compared to a factor of one for the single field (${|{{{{{{\bf{E}}}}}}}_{p}|}^{2}$in Eq. (2)). This arises because of different symmetry permutation associated with degenerate and non-degenerate fields. In other words, the signal and idler experience two times stronger topological tuning via cross-phase modulation (XPM) than that from self-phase modulation (SPM) experienced by the pump. Topological localization of the signal or idler can be broken with half as much pump power using XPM vs. SPM. Further details are provided in Supplementary Note 13. The phase mismatch, Δ*β*, requires a value as close to zero as possible to maximize four-wave mixing phenomena in the topological system. Coupled Eqs. (2)–(4) are used in this manuscript to theoretically calculate the four-wave mixing process occurring in the topological waveguide and analyze their agreement with experiments.

## Results

### Topological waveguide design

**Fig. 2:**
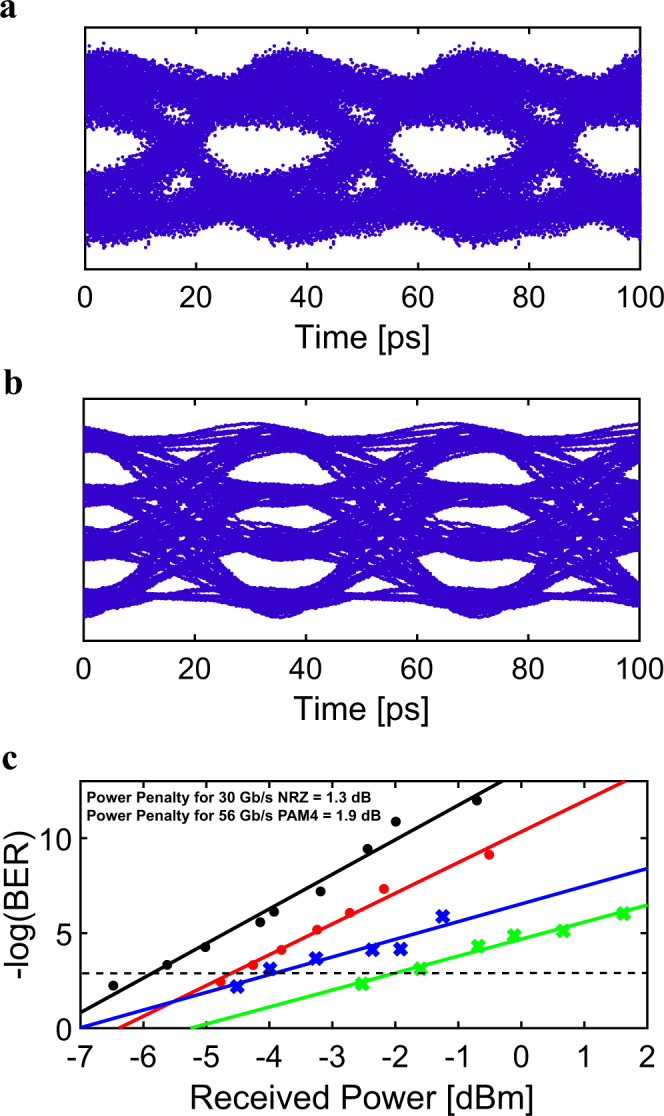
Experimentally measured eye diagrams and BER at the output of the boundary waveguide. **a**, **b** An open eye is observed in both cases for **a** 30 Gb/s NRZ and **b** 56 Gb/s PAM4 data. **c** Experimentally measured plot of –log(BER) as a function of power received at the output of the USRN topological photonic waveguide, for 30 Gb/s NRZ and 56 Gb/s PAM4 data. The black dotted line shows the Forward Error Correction (FEC) threshold at BER = 10^−4^. The measured NRZ BER for the topological waveguide and B2B setup are shown as red and black circles, respectively. The measured PAM4 BER for the topological waveguide and B2B setup is shown as green and blue crosses respectively.

We designed the topological photonic waveguide using ultra-silicon-rich nitride (USRN), which possesses a high nonlinear figure of merit and negligible nonlinear losses at 1550 nm^44–46^. USRN is therefore a prime platform on which to observe Kerr effects occurring in the topological mode. In the SSH photonic system, the hopping amplitude, wave function, and temporal propagation are analogous to coupling coefficients, spatial waveguide modes, and spatial propagation in the waveguide direction, respectively. We implemented the topological boundary state using the structure as shown in Fig. 1b which permits the boundary state to be localized^47^. In our design, 199 waveguides are used to ensure a sufficiently large transversal size in the topological system relative to the size of the localized boundary mode (spans 9 waveguides). The waveguide cross-section is 0.6 μm (*W*) × 0.3 μm (*H*). For this axial waveguide size, the coupling constant between neighboring waveguides is calculated as a function of the gap distance and shown in Fig. 1c. The gap between waveguides is chosen to be *G*_w_ = 0.15 µm and *G*_v_ = 0.25 µm for the narrower and wider gaps, respectively; The respective coupling constants are calculated to be 0.093 and 0.016 µm^−1^ in Fig. 1c for the fundamental transverse-electric mode. We note the importance of the designed values of *G*_w_ and *G*_v_ in providing strong localization to the domain wall, as is discussed in Supplementary Note 1. The sample length is fixed at 4 mm in order to exceed the coupling length and to ensure there is a sufficient interaction length for the observation of nonlinear phenomena. For the sample designed with the parameters, we demonstrate the topological boundary state by measuring output power versus waveguide index. Note that the measured output powers in this manuscript include only the power at the boundary waveguide. Details of the device fabrication are provided in Supplementary Note 2. The output power is well localized at the boundary waveguide indexed to be |0〉 as shown in Fig. 1d. Numerical simulations confirming the strong localization of the light to the boundary waveguide are shown in Fig. 1e and further provided in Supplementary Note 3. Supplementary Note 5 shows the measured output power as a function of waveguide index in a trivial (non-topological) waveguide, where it is observed that light is not localized to the boundary waveguide but rather, spreads to adjacent waveguides considerably. We note from Fig. 1d, the strong localization of the topological mode to waveguide |0〉 measured over a wide wavelength range. This feature is especially critical when implementing the topological waveguide for bandwidth-sensitive applications such as high-speed transmission and nonlinear light generation, both of which are studied in this paper. Further characterization of the linear transmission properties of the topological waveguide is performed using 30 Gb/s NRZ and 56 Gb/s PAM4 high-speed data (experimental details in Supplementary Note 6). Figure 2a, b shows the measured eye diagram for 30 Gb/s NRZ and 56 Gb/s PAM4 high-speed data measured at the output of the 4 mm topological waveguide. Both eyes are observed to be clear and open. Figure 2c shows the measured bit error rate (BER) as a function of received power, as well as the back-to-back (B2B) BER readings. At the forward error correction (FEC) threshold for error-free transmission (BER = 10^−4^, black dotted line), a low power penalty of 1.3 and 1.9 dB for 30 Gb/s NRZ and 56 Gb/s PAM4 data, respectively, is observed. The extrapolated power penalty for 30 Gb/s NRZ data at a BER of 10^−12^ is 1.8 dB. We note that other topological photonic waveguides used for high-speed communications were previously documented to be bandwidth limited, restricting the data transfer rate to 16 Gb/s (at FEC threshold)^48^. The high-speed characterization results confirm the good linear transmission properties of the USRN topological photonic waveguide and demonstrate their suitability for high-speed data transmission, importantly not being bandwidth limited.

### Parametric gain and wavelength conversion in USRN topological waveguides

Next, we perform degenerate four-wave mixing experiments in the USRN topological waveguides with a length of 4 mm. The pump pulses are derived from a fiber laser with a 20 MHz repetition rate with a temporal pulse width of 5 ps. The signal is derived from a tunable continuous wave laser. The pump and signal are combined using a wavelength division multiplexer and output spectra are measured using an optical spectrum analyzer. In the regime where dispersion is strong enough to reduce the forbidden band barrier corresponding to |*w*−*v*|, the topological boundary states could be vulnerable to sources of noise or fabrication error. When the pump, signal, and idler propagate within the waveguide, it is experimentally observed that the spectrum changes minimally. We can therefore confirm that the topological boundary state is intact. In the absence of other perturbations, the generated signal and idler will remain guided in the boundary waveguide in spite of any existing randomness in the distribution of coupling coefficients in the dimerized topological waveguide array. Thus, the demonstrated waveguide array serves as a topological vessel for the nonlinear effects inherent in USRN to take place.

**Fig. 3:**
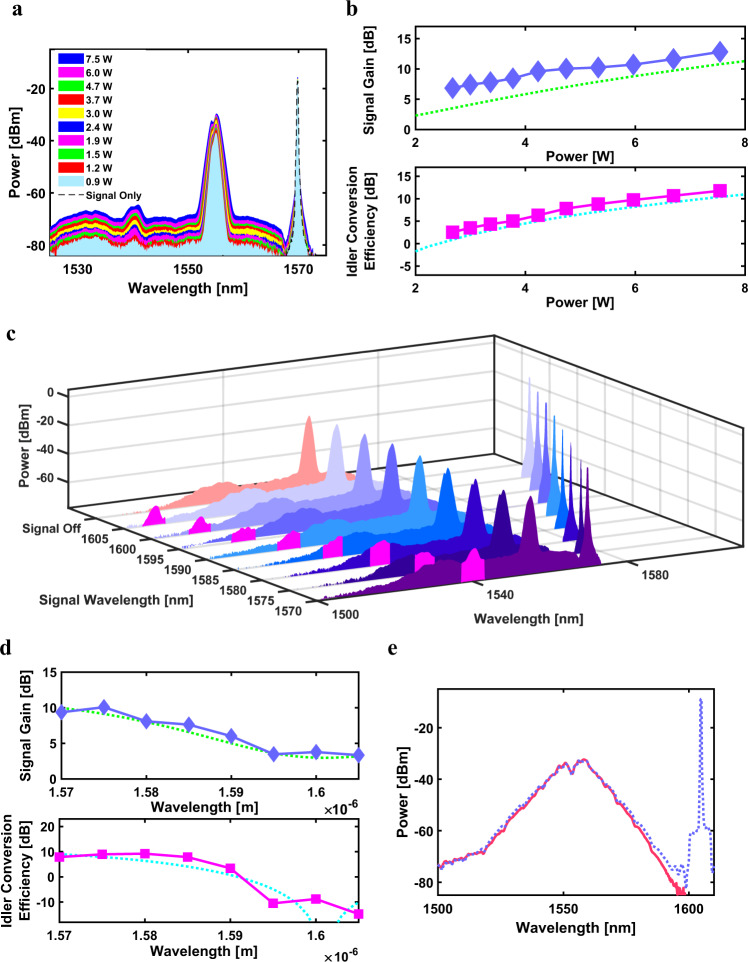
Experimentally measured optical parametric wavelength conversion in the topological boundary states. **a** Four-wave mixing spectrum for 5 ps pump pulses and as a function of pump peak power. The different spectra represent increasing pump peak powers from 0.9 to 7.5 W. Signal sidebands characteristic of parametric gain are shown to develop when the pump is on. **b** Measured (blue diamonds) and calculated (green dashed line) signal gain as a function of peak power calculated using the coupled equations. Measured (fuchsia squares) and calculated (cyan dashed line) idler conversion efficiency as a function of pump peak power. **c** Four-wave mixing spectra as a function of signal wavelength. The signal spectrum with the pump off is shown as the black dashed line. The locations of the generated idlers are shown in fuchsia. **d** Measured (blue diamonds) and calculated (green dashed line) signal gain as a function of signal wavelength. Measured (fuchsia squares) and calculated (cyan dashed line) idler conversion efficiency as a function of signal wavelength. Calculations were performed using the coupled equations. **e** Four-wave mixing spectra when 1 ps pulses are used as the pump. The pump spectrum with the signal off is the red solid line, whereas the spectrum where both pump and signal are on is shown as the blue dotted line. In this case, no idler is observed.

First, the idler spectrum induced by four-wave mixing is experimentally observed as pump peak power is increased. For these experiments, the pump and signal wavelengths are fixed at 1.555 and 1.57 μm. It may be observed in Fig. 3a that the idler spectrum appears at ${\lambda }_{{{{{{\rm{i}}}}}}}=1/\left(2{/\lambda }_{{{{{{\rm{p}}}}}}}-1/{\lambda }_{{{{{{\rm{s}}}}}}}\right)$ by energy conservation. As the peak power of the pump is increased from 0.9 to 7.5 W, the conversion efficiency of the idler is observed to increase from 2.7 to 11.6 dB (Fig. 3b). Increasing sideband levels are also observed to develop around the signal when the pump is on, characteristic of parametric amplification. The parametric gain in the signal increases from 6.8 to 12.8 dB with increasing pump powers. Broadening in the pump spectrum from self-phase modulation occurs as the pulses propagate through the topological waveguide. We note further that when a 4 mm trivial waveguide (*G*_w_ = *G*_v_ = 0.25 µm) was used for the experiments under the same pump and signal configuration, no idler could be observed, indicating that the conversion efficiency was substantially lower. Further details may be found in Supplementary Note 12.

The impact of varying the signal wavelength while fixing the pump peak power and wavelength at 7.5 W and 1.555 μm, respectively, are investigated. Figure 3d shows the spectra generated at the output of the topological waveguide where the idler is observed to shift according to the relative detuning between pump and signal. Considering the phase matching requirements in degenerate four-wave mixing, an increased pump-probe detuning in the regime of the experiment is expected to lead to reduced phase matching and a concomitant decrease in the overall signal gain and idler conversion efficiency. The signal gain and idler conversion efficiency as a function of wavelength are shown in Fig. 3c, where it is observed that the signal gain and idler conversion efficiency decreases as the pump-signal detuning is increased. The theoretical signal gain and idler conversion efficiency as a function of peak power and wavelength are further calculated using the coupled equations and shown in Fig. 3b, d. We achieve good agreement between the experimentally measured and theoretically calculated values.

We next study the impact of a strong pump in creating a non-negligible increase in the local refractive index of the boundary waveguide to induce chiral symmetry breaking in the topological waveguide. To shed light on this phenomenon, we compare the four-wave mixing results when using a pump pulse width of 5 and 1 ps to achieve greater peak power. When utilizing 1 ps pump pulses with a higher peak power of 29.5 W, no idler is observed for all signal wavelengths studied. Figure 3e shows the measured output spectra when a 1 ps pump co-propagates with a signal at a wavelength of 1.605 μm; No idler can be observed. We postulate that the absence of four-wave mixing observed for the higher peak power likely occurs because the optical power guided in the boundary state is reduced as a result of the Kerr-induced nonlinear perturbation of the topological boundary state, which causes a breaking in chiral symmetry; We conduct a series of further optical characterizations to confirm this hypothesis.

**Fig. 4:**
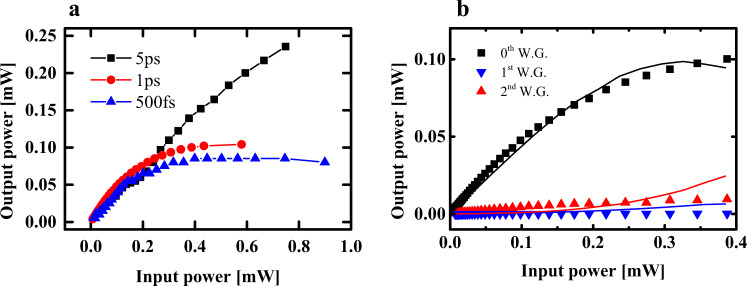
Experimentally observed output power saturation in the topological boundary waveguide. **a** The output power versus input power at different temporal pulse widths of 5, 1 ps, and 500 fs. **b** The output power of 1 ps pulses at the boundary waveguide and the 1st and 2nd neighboring waveguides, where the points are measured data of output powers vs. input powers and solid lines correspond to theoretical results calculated using the nonlinear Schrödinger equation.

First, we monitor the output power as a function of input power to study whether the optical power guided in the topological mode experiences a decrease when the input peak power coupled into the topological waveguide is increased. A decrease in the transmitted power would indicate that the topological boundary state is not well localized in the boundary waveguide. In the presence of chiral symmetry (absence of nonlinear perturbation), the odd waveguides would have a zero amplitude. A break in the topological boundary state manifests itself as an increase in the optical amplitude guided in the nearest neighbor (odd-numbered) waveguide. Figure 4a shows the output power of the topological boundary waveguide as a function of average input power for the 5 ps pump beam, where a linear relationship is observed.

However, the relationship between output and input power for the 1 ps and 500 fs pulses where input peak powers are 5× and 10× larger at the same average power, reveals a different trend. Specifically, the output power of the topological boundary waveguide is observed to saturate at an input average power of 0.42 and 0.30 mW, respectively (Fig. 4a). We note further that this observed saturation cannot be due to nonlinear absorption, as significantly higher peak powers have previously been used in USRN waveguides without any observation of output power saturation^49,50^. The output power in the boundary waveguide and the next-neighbor waveguide when 1 ps pulses are used is measured in Fig. 4b. It is observed that the power measured at the output of the nearest-neighbor waveguide increases with the input power. This observed phenomenon together with the saturation in output power in the boundary waveguide indicates that the Kerr nonlinear effect can control the topological mode by effectively breaking the chiral symmetry in the waveguide lattice. We further calculate the output power at waveguide |0〉 and waveguide |+1〉 corresponding to the boundary waveguide and the nearest neighbor waveguide using the nonlinear Schrödinger equation. The result is shown in Fig. 4b. The onset of output power saturation is observed to occur when the input average power of the 1 ps pulses approaches 0.6 mW, similar to that observed experimentally in Fig. 4b.

**Fig. 5:**
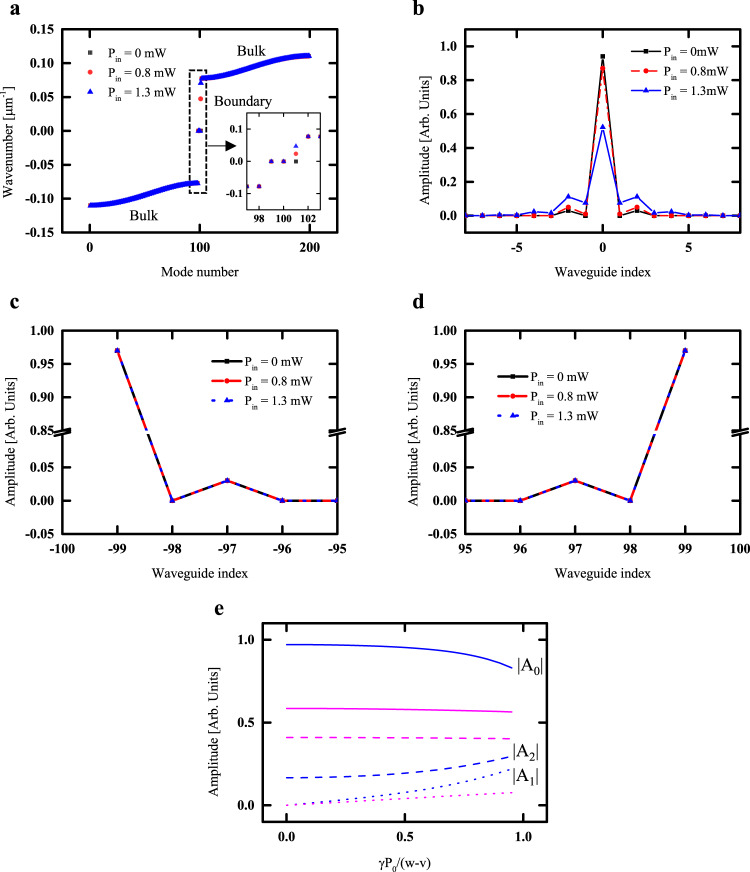
The topological boundary state tuned by the Kerr nonlinearity. **a** Band diagram of the topological waveguide for different input power. Distribution of the wave function for the **b** boundary mode, **c** left-hand edge mode, and **d** the right-hand edge mode. **e** Amplitude variation induced by the Kerr perturbation for *v*/*w* = 0.17 (Blue) and *v*/*w* = 0.7 (Magenta).

We postulate that the observed saturation in the output power is induced by a nonlinear Kerr perturbation to the boundary state. The higher peak power of the 1 ps pulses induces a substantial distortion in the propagation phase, $\gamma {P}_{0}$, large enough to go over the barrier of the forbidden band. To analyze this hypothesis, we perform further analyses using the discretized model to study the potential for Kerr-based tuning of the topological boundary mode. Details of the discretized model are provided in Supplementary Note 11. We show in Fig. 5a, the wavenumber vs. mode number at various powers. Our system is defined as having one boundary mode corresponding to mode number 101, residing between bulk topology 1 and 0 by bulk-boundary correspondence^51,52^. The boundary mode is the zero modes in the chirally symmetric system and the boundary state will remain locked at the zero modes even if other perturbations are present, as long as these perturbations do not disrupt the system’s chiral symmetry. Nonlinear Kerr perturbations however are not classified as perturbations that preserve chiral symmetry. In the regime where perturbations from optical nonlinearity are non-negligible and the nonlinear refractive index has a positive value, the boundary mode approaches the upper edge of the forbidden band when the power is sufficiently large. In this case, the barrier becomes too shallow to keep the boundary state localized to the boundary waveguide. In line with the odd waveguides in the unperturbed SSH system possessing a vanishing amplitude, this de-localization of the boundary state will manifest itself as a finite amplitude in the odd waveguides, consistent with the observation of increasing optical amplitude in the nearest-neighbor waveguide discussed in Fig. 5b. In this case, the boundary state is no longer strongly localized in the boundary waveguide as shown in Fig. 5b. For a system with chiral symmetry, the amplitude in |±1〉 should be zero. Summarily, a sufficiently large Kerr perturbation will result in: (i) a reduction in the amplitude of the boundary waveguide |0〉, (ii) a non-zero amplitude in the waveguide |±1〉, and (iii) an increase in the amplitude in the waveguide |±2〉. As shown in Fig. 5e, the degree of de-localization induced by the Kerr nonlinear effect varies with the magnitude of *v*/*w*. In Fig. 5e, the blue lines represent *v*/*w* = 0.17 corresponding to our designed topological waveguide whereas magenta lines correspond to *v*/*w* = 0.7. In the figure, solid lines, dotted lines, and dashed lines denote the amplitudes at the 0th, 1st, and 2nd waveguides, respectively. Strong de-localization of wavefunction induced by the Kerr nonlinear effect occurs for a small value of *v*/*w* < 0.5 and manifests as a reduced amplitude at the boundary waveguide (0th waveguide) and increased amplitude at the other waveguides (1st and 2nd). It is further observed that the de-localization induced by the Kerr effect is less pronounced when 0.5 < *v*/*w* < 1. In this case, the Kerr perturbation results in an amplitude reduction at the boundary and even-numbered waveguides but an increased amplitude at the odd-numbered waveguides. These observations indicate that the de-localizing effect induced by the Kerr nonlinear perturbation is stronger for a well-localized boundary state as shown in Fig. 5e.

Analogously, the Kerr perturbation may be viewed as an onsite potential commonly encountered in topological states of matter in condensed matter physics: The wave function of the topological mode will experience an overall broadening effect which becomes increasingly pronounced as the magnitude of the on-site potential increases (Supplementary Note 1). In the topological waveguide based on the SSH model with a domain wall, the eigenmodes for the two edge states corresponding to modes 99 and 100 exist in the forbidden barrier but the states are localized at the two edges far from the boundary waveguide as shown in Fig. 5c, d. The amplitude distribution in the left and right edge states will remain unchanged even in the presence of the Kerr perturbation because the boundary waveguide where the perturbation is applied is far from the edge waveguides.

## Discussion

We note that amongst recent advancements in studies of nonlinear effects on topology-driven light, two bodies of work are particularly interesting in the context of the work reported here. Firstly, previous theoretical findings postulated that nonlinearities could induce topological phase transitions, for example, to achieve optical isolation or undergo topological mode transitions^53–55^. Our experimental findings and analyses showcase the ability to tune the topological mode’s degree of localization and confirm that Kerr nonlinearities may indeed present a pathway towards inducing chiral symmetry breaking and control of the topological boundary mode at relatively low powers. Given the accessibility of this topological tuning mechanism, one could envisage its pertinence towards future applications in topological modulators, topological switches, topological saturable absorbers, and power stabilizers. Secondly, recent noteworthy advancements made in the nonlinear generation of light involved harmonic generation in topological photonic nanostructures^39,56,57^, whereas work in topological solitons involves nonlinear interactions acting on a single optical field rather than interactions between co-propagating optical fields. Ref. 57 in particular leverages the spin-Hall effect in a higher-order topological photonic crystal in silicon to enhance the harmonic generation, generating a conversion efficiency of 1.8 × 10^−7^. While the reported conversion efficiency is 80 dB lower compared to that observed in our SSH-based optical parametric amplifier, some similarities exist. Our demonstration of topological nonlinear parametric gain relies on intrinsic material nonlinearity, with an advantage in USRN vs. silicon being the absence of two-photon absorption at telecommunications wavelengths. Furthermore, the SSH system studied in our work provides stronger modal confinement within the nonlinear material compared to previously reported topological corner states. Nevertheless, we note that the two topological systems possess different topological phases, dimensions, and modal profiles, each with its own unique merits. Our work is further distinguished from prior work, relying instead on the mixing of pump and probe photons within a topological edge state, in a process involving momentum conservation, allowing flexible topological light amplification and generation at frequencies that are determined by the relative difference between the pump and probe frequencies. Conversely, light generation involving harmonic generation has their generated wavelengths limited to the harmonic of the pump wavelength. The experimental demonstration of parametric gain and wavelength conversion in the regime where chiral symmetry is preserved is facilitated by the strong localization of the zero modes to the topological boundary and provides evidence that the USRN topological waveguide serves as a potent channel in which efficient, low power, nonlinear light generation, and amplification may occur. The low powers required to tune the topological boundary mode and for topological parametric wavelength conversion underscore the uniqueness and practicality of the demonstrated topological photonic waveguide based on the SSH model with a domain wall. Control of the transmittance and optical parametric gain at a boundary waveguide gives us further insight into how the tuned topological mode may impact parametric processes. The ability to tune the topological mode using nonlinear mechanisms further provides control on femtosecond time scales characteristic of the Kerr effect. The efficient parametric light amplification and generation demonstrated in the USRN topological photonic waveguide make us optimistic about the silicon photonics route toward driving topological photonics closer to commercial applications. This work serves as the foundation on which further design advancements could be made toward topological photonic parametric amplifiers, parametric oscillation, ultrafast topological switches, modulators for high-speed data, and other important Kerr-based topological silicon photonics applications, as well as new methods for their control through nonlinear topological phenomena.

## Supplementary information

Supplementary Information File (PDF Format)

## Data Availability

Data from this study are available from the corresponding author upon reasonable request.
