# Supplementary material for: A topological nonlinear parametric amplifier
Source: Nat Commun. 2022 Nov 24;13:7218. doi: 10.1038/s41467-022-34979-y (PMC9700758; doi:10.1038/s41467-022-34979-y)
Supplement: Supplementary file 1 — Supplementary Information File (PDF Format) [file 41467_2022_34979_MOESM1_ESM.pdf]

# A topological nonlinear parametric amplifier

Byoung-Uk Sohn, [1] Yue-Xin Huang, [2] Ju Won Choi, [1] George F. R. Chen, [1] Doris K. T. Ng, [3] Shengyuan A. Yang, [2] and Dawn T. H. Tan [1,3,*]

[1]Photonics Devices and System Group, Singapore University of Technology and Design, Singapore, 487372, Singapore
[2]Research Laboratory for Quantum Materials, Singapore University of Technology and Design, Singapore, 487372, Singapore
[3]Institute of Microelectronics, A*STAR, 2 Fusionopolis Way, #08-02, Innovis Tower, Singapore 138634, Singapore

* dawn_tan@sutd.edu.sg

## Supplementary Information

## I. Supplementary Note 1: SSH model with a domain wall

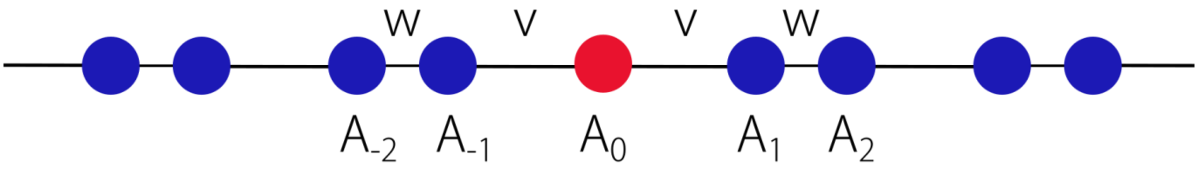


**Supplementary Figure 1** Su-Schrieffer-Heeger (SSH) model with a domain wall at the center. The amplitude $A_i$ denotes the amplitude of the topological mode at each site (waveguide). $v$ and $w$ are the couplings across long and short gaps, respectively. We should have $v < w$. A topological mode exists and is localized at the domain wall around the central waveguide (red).

As stated in the main text, the system we consider can be mapped to an SSH model with a domain wall at the center, as depicted in Supplementary Figure 1. A topological mode must exist and be localized at the domain wall. The corresponding 1D effective Hamiltonian when the basis is ordered as $|-n\rangle, \cdots, |-1\rangle, |0\rangle, |1\rangle, \cdots, |n\rangle$, where the index $|\mathrm{i}\rangle$ denotes

the $i^{\text{th}}$ waveguide. The $(2n+1)\times(2n+1)$ matrix for $\mathbf{K}$ has the following form. The only non-vanishing elements are $(i=1,\cdots,2n)$

$$\mathbf{K}_{i,i+1}=\mathbf{K}_{i+1,i}=\begin{cases}\frac{1}{2}\left[1+(-1)^{i+n}\right]v+\frac{1}{2}\left[1-(-1)^{i+n}\right]w,\ i\leq n\\ \frac{1}{2}\left[1-(-1)^{i+n}\right]v+\frac{1}{2}\left[1+(-1)^{i+n}\right]w,\ i\geq n+1\end{cases}\tag{1}$$

Clearly, the system has an inversion symmetry with respect to the center, which can be expressed as,

$$\mathbf{I}_{i,j}=\begin{cases}1\,,\ if\ \ i+j=2n+2\\ 0, \qquad\qquad otherwise\end{cases}\tag{2}$$

One can easily check that it commutes with the effective Hamiltonian $[\mathbf{I},\mathbf{K}]=0$. Moreover, the system also preserves a chiral symmetry, which reads,

$$\mathbf{S}_{i,j}=\begin{cases}(-1)^{i-n-1}, \quad if\ i=j\\ 0, \qquad otherwise\end{cases}\tag{3}$$

such that $\{\mathbf{K},\mathbf{S}\}=0$, meaning that a state $\mathbf{A}$ with an eigenvalue E must be accompanied by another state $\mathbf{SA}$, with a negative eigenvalue $-E$. It follows that the eigen-spectrum is symmetric about zero and the topological mode must have a zero eigenvalue.

Moreover, the chiral symmetry dictates that the amplitude of the topological mode on odd sites must be zero. For example, let us consider a system with 9 sites, and express the topological mode as,

$$\mathbf{A}^{topo}=(A_{-4},A_{-3},A_{-2},A_{-1},A_0,A_1,A_2,A_3,A_4).\tag{4}$$

In view of the chiral symmetry, the state $\mathbf{SA}^{\text{topo}}$ is also an eigenstate of $\mathbf{K}$ with zero eigenvalue. Notice that there is no additional degeneracy and all the parameters are real, then we should have $\mathbf{A}=\mathbf{SA}$. This condition leads to:

$$\mathbf{A}^{topo} = (A_{-4}, 0, A_{-2}, 0, A_0, 0, A_2, 0, A_4), \tag{5}$$

where the amplitude on the odd waveguide is suppressed. Besides, the inversion operator is anti-diagonal, requiring the wave-function be symmetric about the center. Hence,

$$\mathbf{A}^{topo} = (A_4, 0, A_2, 0, A_0, 0, A_2, 0, A_4). \tag{6}$$

The remaining amplitudes are constrained by the secular equation $\mathbf{KA} = 0$. One can find that these amplitudes decay exponentially away from the domain wall. The underlying physics can be understood easily. For example, the vanishing amplitude $A_1$ is the consequence of the destructive interference of the hopping from neighboring sites, i.e., hopping from $A_2$ and hopping from $A_0$. Thus, we have,

$$vA_0 + wA_2 = 0 \Rightarrow A_2 = -\frac{v}{w}A_0. \tag{7}$$

In the similar way, the destructive interference in the third waveguide gives,

$$A_4 = -\frac{v}{w}A_2. \tag{8}$$

The results can be readily generalized to more sites, and we can obtain,

$$A_i^{topo} = A_{2n+2-i}^{topo} = \frac{1}{2}\alpha_0 \left[1 - (-1)^{i+n}\right] q^{\frac{n+1-i}{2}}, \qquad q = -v/w \tag{9}$$

Where $i = 1, \cdots, n+1$, and $\alpha_0 = \sqrt{\frac{1-q^2}{1+q^2}}$ is the normalization factor.

For a sufficient large nonlinear refractive index, the onsite potential cannot be ignored. Since the nonlinear effect on the central waveguide is the largest, we consider a simple case where a perturbation term $\mathbf{V} = \epsilon\, |0\rangle\langle 0|$ is added to the central site. This potential destroys the chiral symmetry S and shift energy of the zero mode. By treating $\mathbf{V}$ as a perturbation, the first order of the energy correction has the form of,

$$\varepsilon' = \frac{1 - q^2}{1 + q^2}\epsilon, \tag{10}$$

linear with the onsite potential $\epsilon$. Insert the energy correction back into the eigenvalue equation, we obtain the amplitude of the topological mode on the odd site waveguides up to second order as:

$$A_{2n+1} = \frac{\epsilon \alpha_0}{w(1 + q^2)} q^{|n|+1}, \tag{11}$$

and on the even sites as:

$$A_{2n} = \alpha_0 \left[1 - \frac{1 + 2\alpha_0}{w^2(1 + q^2)^3} q^2 \epsilon^2\right] q^{|n|}. \tag{12}$$

Therefore, from these two equations, we observe the following:

(i) An increase in the magnitude of *w* relative to *v* leads to a stronger localization of the topological mode's spatial distribution to the central waveguide (Supplementary Figure 2).

(ii) The introduction of an onsite potential (akin to a Kerr perturbation) at the central waveguide leads to a reduction in the amplitude of the central waveguide and all even waveguides and an amplitude increase in the odd waveguides. In other words, the localization to the central waveguide decreases and overall, the topological mode distribution broadens (Supplementary Figure 3).

(iii) The broadening in the topological mode distribution becomes more pronounced as the magnitude of onsite potential (akin to a Kerr perturbation) increases (Supplementary Figure 4).

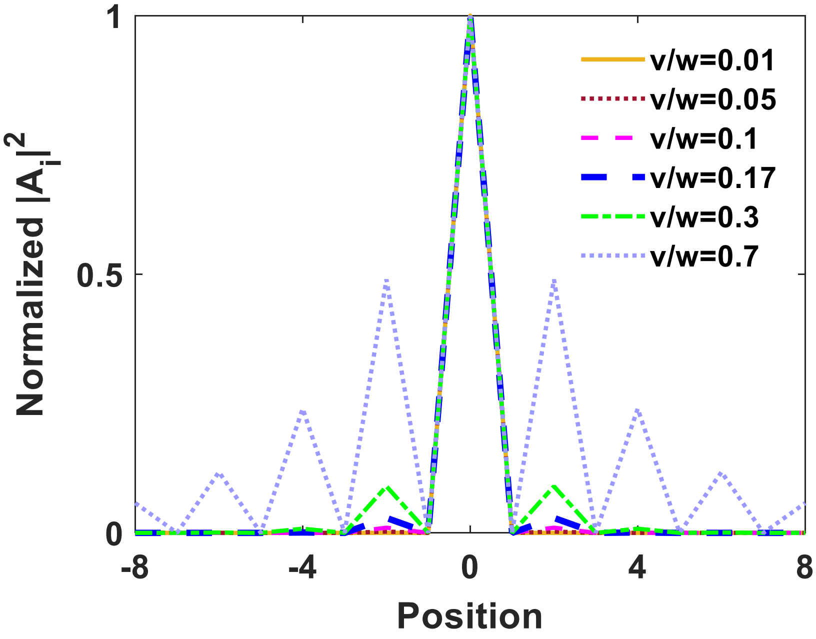


**Supplementary Figure 2** Spatial distribution of the topological mode as a function of *v*/*w* for zero onsite potential. The central peak of $|A_i|^2$ is normalized to unity. Here, we take $w$ = 1.0 and vary $v$ to study the impact of *v*/*w*. It is observed that the mode is strongly localized to the central waveguide (Position 0): All odd sites have zero amplitude, and all even sites other than Position 0 have negligible amplitude. The amplitude at the even sites is observed to increase when *v*/*w* increases, consistent with a reduction in localization.

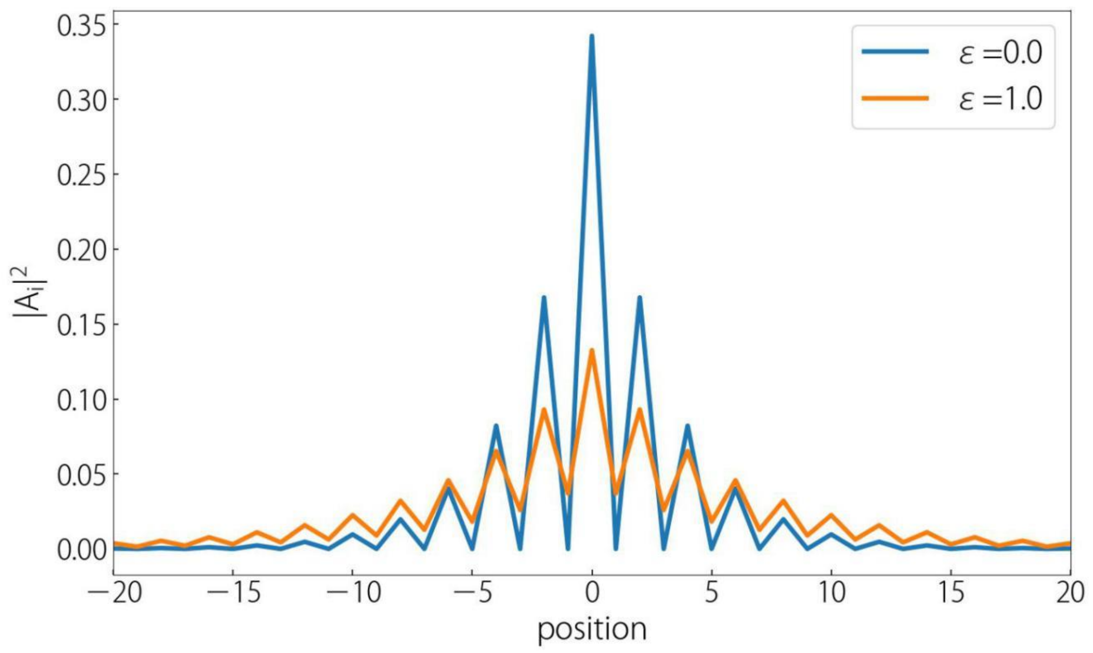


**Supplementary Figure 3** Spatial distribution of the topological mode with different onsite potential. The onsite potential at the central site increases the amplitude in the odd-numbered waveguides and broadens the topological mode. Here we take $w = 1.0$ and $v = 0.7$ in the model.

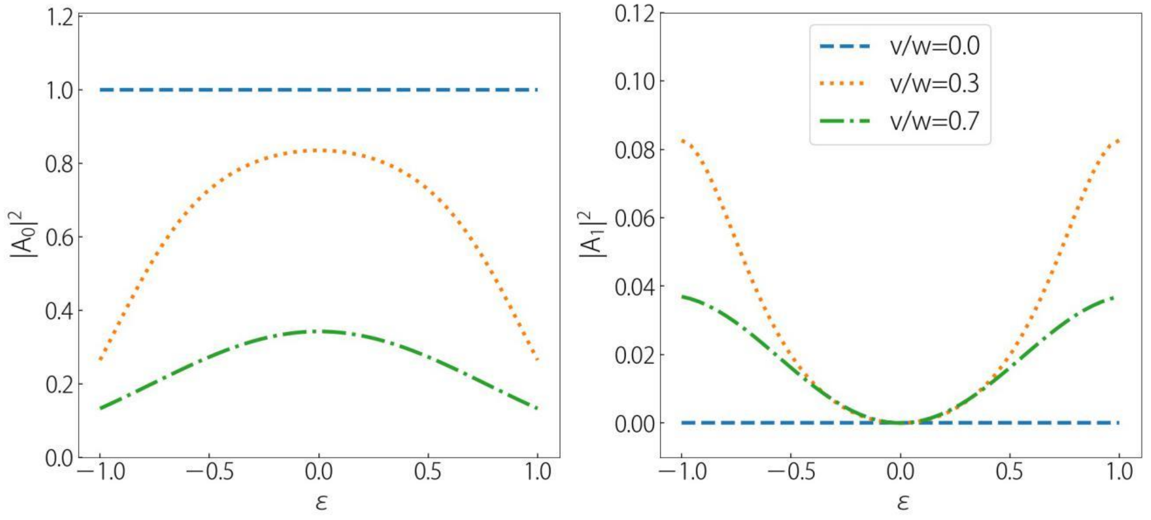


(a) (b)

**Supplementary Figure 4** Amplitude of $A_0$ and $A_1$ with different parameters $v/w = 0, 0.3, 0.7$. From the figures, both **a**, $|A_0|^2$ and **b**, $|A_1|^2$ change along with the onsite potential in order of $\epsilon^2$ (see Eq (11) and (12) for the explicit expressions).

## II. Supplementary Note 2: Fabrication details

The topological waveguide is fabricated on the USRN platform. Supplementary Figure 5 (a) shows the fabrication process undertaken. 300 nm of USRN is first deposited on a 3 μm thick thermal $SiO_2$ on Si substrate using inductively coupled chemical vapor deposition at a low process temperature of 250°C. The waveguide is first spin-coated with electron-beam resist and patterned using electron-beam lithography. Inductively coupled plasma etching is performed to define the device structure. The residual resist is removed before 2 μm of $SiO_2$ overcladding is deposited using atomic layer deposition and plasma enhanced chemical vapor deposition. An additional scanning electron micrograph of the fabricated USRN topological waveguide showing the domain wall is shown in Supplementary Figure 5 (b). The USRN platform has a linear and nonlinear refractive index of 3.1 and $2.8 \times 10^{-13}$ $cm^2 W^{-1}$ respectively [1-3]. USRN has a bandgap of 2.1 eV and the two-photon absorption edge is located at 1.2 μm [1]. Two-photon absorption is therefore absent at the wavelengths used in the experiments.

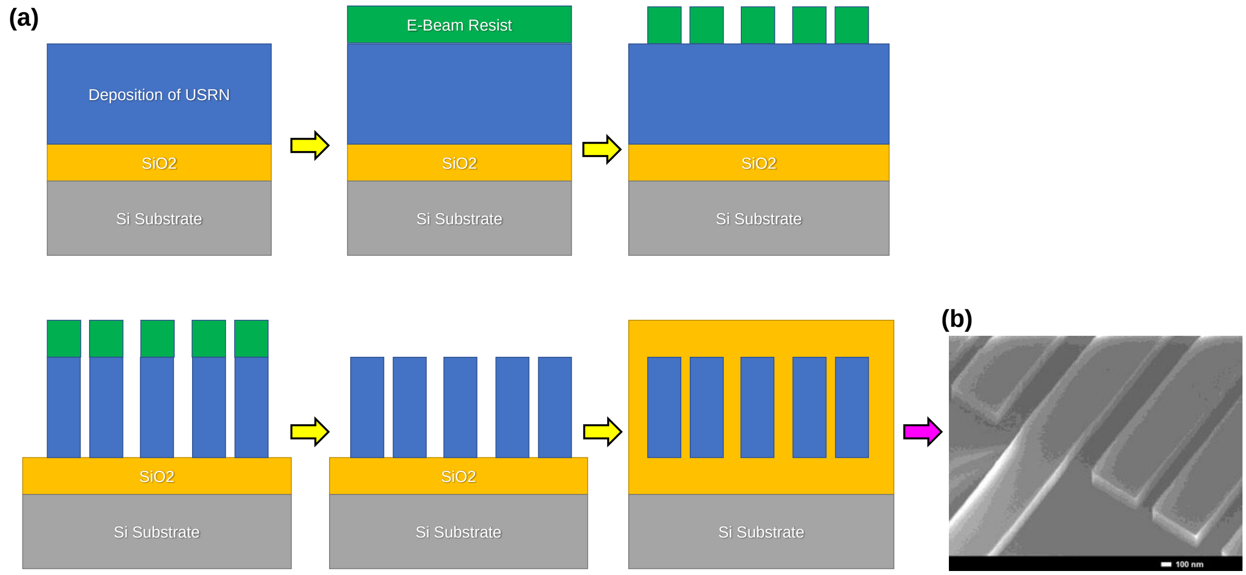


**Supplementary Figure 5 a**, Fabrication process steps for the USRN topological photonic waveguide. **b**, Scanning electron micrograph of the fabricated device showing the domain wall where the topological boundary state resides.

## III. Supplementary Note 3: Numerical simulations for the propagation of light in the topological waveguide

Finite difference time domain simulations are performed to study the propagation of light within the USRN topological waveguide. Supplementary Figure 6 (b) shows the time-dependent evolution of the optical field as it propagates through the waveguide. It is observed that most of the light is localized at the boundary waveguide ($|0\rangle$), the amplitude at the odd waveguides is negligible, and a very small amplitude exists in waveguides $|\pm2\rangle$. The trivial waveguide array where $G_v = G_w = 0.25$ μm is further shown in Supplementary Figure 6 (a), where it is observed that the light propagation is not confined to the boundary waveguide but rather, spreads out akin to spatial diffraction, as it traverses the length of the waveguide array.

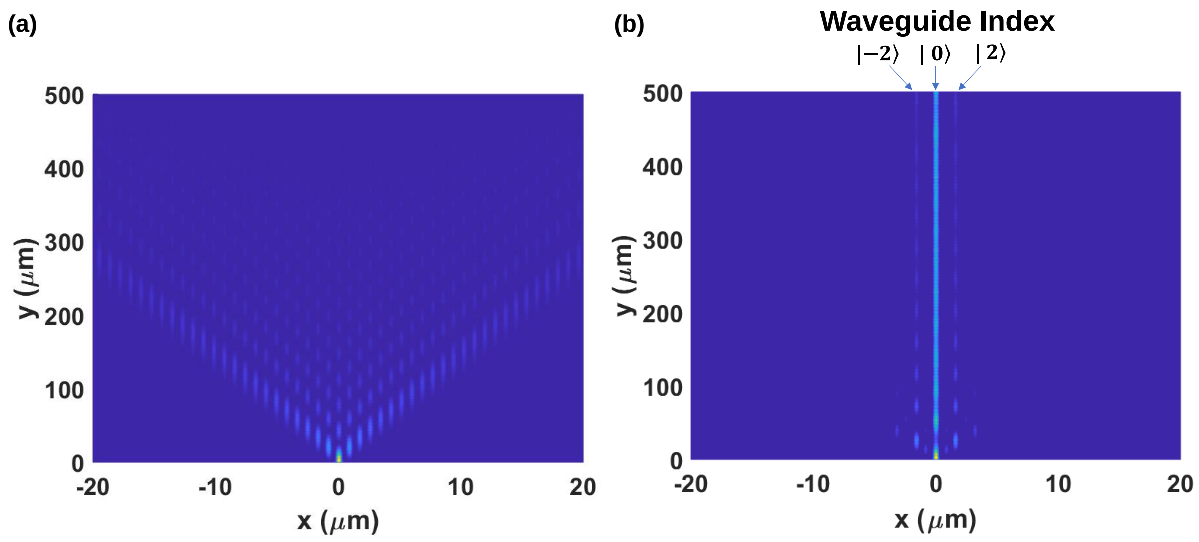


**Supplementary Figure 6** Numerically simulated light propagation within the **a**, trivial waveguide array and **b**, USRN topological waveguide. Here, *y* is the direction of propagation and *x* is the transverse direction. The locations of waveguides $|-2\rangle$, $|0\rangle$ and $|2\rangle$ are shown.

## IV. Supplementary Note 4: Experimental details for linear characterization of the USRN topological photonic waveguide

To experimentally characterize the USRN topological photonic waveguide, a wavelength tunable continuous-wave laser is used as the source. A fiber-based polarizer is used to select the transverse-electric mode prior to coupling the light into the device. The device is mounted on a high-resolution motorized stage to align the output waveguides with a tapered fiber. The output power at each of the ports is measured using a power meter. Coupling into the topological waveguide is facilitated with a tapered fiber to maximize excitation of the boundary mode. The fiber-waveguide coupling loss is 7 dB.

## V. Supplementary Note 5: Linear characterization of a trivial USRN waveguide.

A trivial USRN waveguide with $G_v = G_w = 0.25$ μm and waveguide cross-section of 0.6 μm (*W*) × 0.3 μm (*H*) was fabricated to compare its light guiding properties with the SSH waveguide. Using the experimental setup described in Supplementary Note 4, the output from waveguides $|-4\rangle$ to $|4\rangle$ is measured. Supplementary Figure 7 shows the optical transmission for the trivial waveguide. In the trivial waveguide, it is observed that light is not localized to the boundary waveguide. Rather, the light spreads, with substantial amplitude propagating in the adjacent waveguides, further demonstrating that the SSH waveguide structure provides a key advantage over the trivial waveguide by enabling topologically induced localization of light to the boundary waveguide.

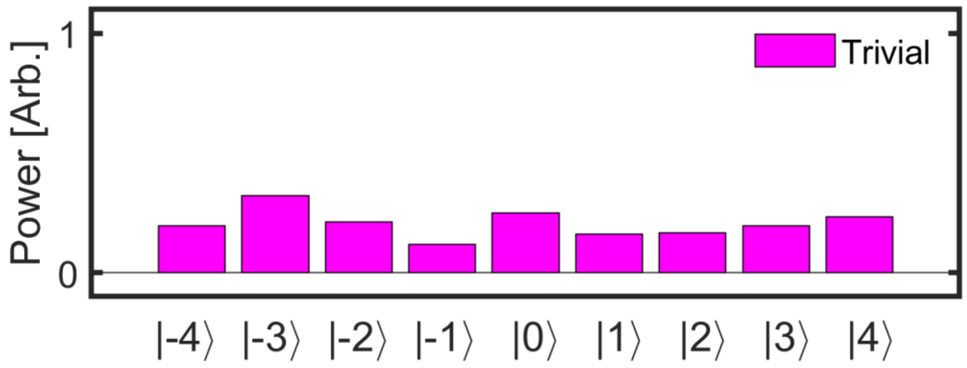


**Supplementary Figure 7** Linear characterization of trivial waveguides ($G_v = G_w = 0.25$ μm) showing the power at waveguide indices $|-4\rangle$ to $|4\rangle$ are shown in the x-axis.

## VI. Supplementary Note 6: Details of the high-speed characterization experiments

To further study the linear transmission properties of the USRN topological photonic waveguide, further optical characterization was performed using high-speed testing. A Pseudo Random Binary Sequence of $2^{31}$-1 (PRBS31) in used for the generation of bit patterns. The modulation formats used were 30 Gb/s Non-Return-Zero-On-Off-Keying (NRZ-OOK) and 28 GBaud/s Pulse Amplitude Modulation 4-Level (PAM4), equivalent to

a bit rate of 56 Gb/s. We note that both NRZ and PAM4 modulation formats utilize direct detection and are widely deployed for high-speed data movement hardware commercialized by silicon photonics companies, for example those within the PSM4 and 100G Serial Lambda multisource agreements [4,5].

The experimental setup is shown in Supplementary Figure 8. A Mach Zehnder Optical Transmitter modulates a Continuous Wave (CW) Laser operating at 1550.12 nm using the PRBS31 patterns generated by a Pattern Generator from the Bit Error Rate Tester (BERT). The 1550.12 nm carrier wavelength used (Optical Frequency = 193.40 THz) corresponds to one of the 100 GHz DWDM wavelengths within the G694.1 ITU Grid standard. The modulated output was then polarized and amplified before being launched into the topological waveguide using a tapered fiber in the quasi-TE configuration. The output of the topological waveguide is amplified using a low noise amplifier before being fed into a photoreceiver for Optical-To-Electrical conversion. The converted electrical data signal was analyzed using a digital sampling oscilloscope (DSO) to retrieve the eye diagram. The bit error rate was also characterized using the BERT Receiver. The setup for PAM4 is the

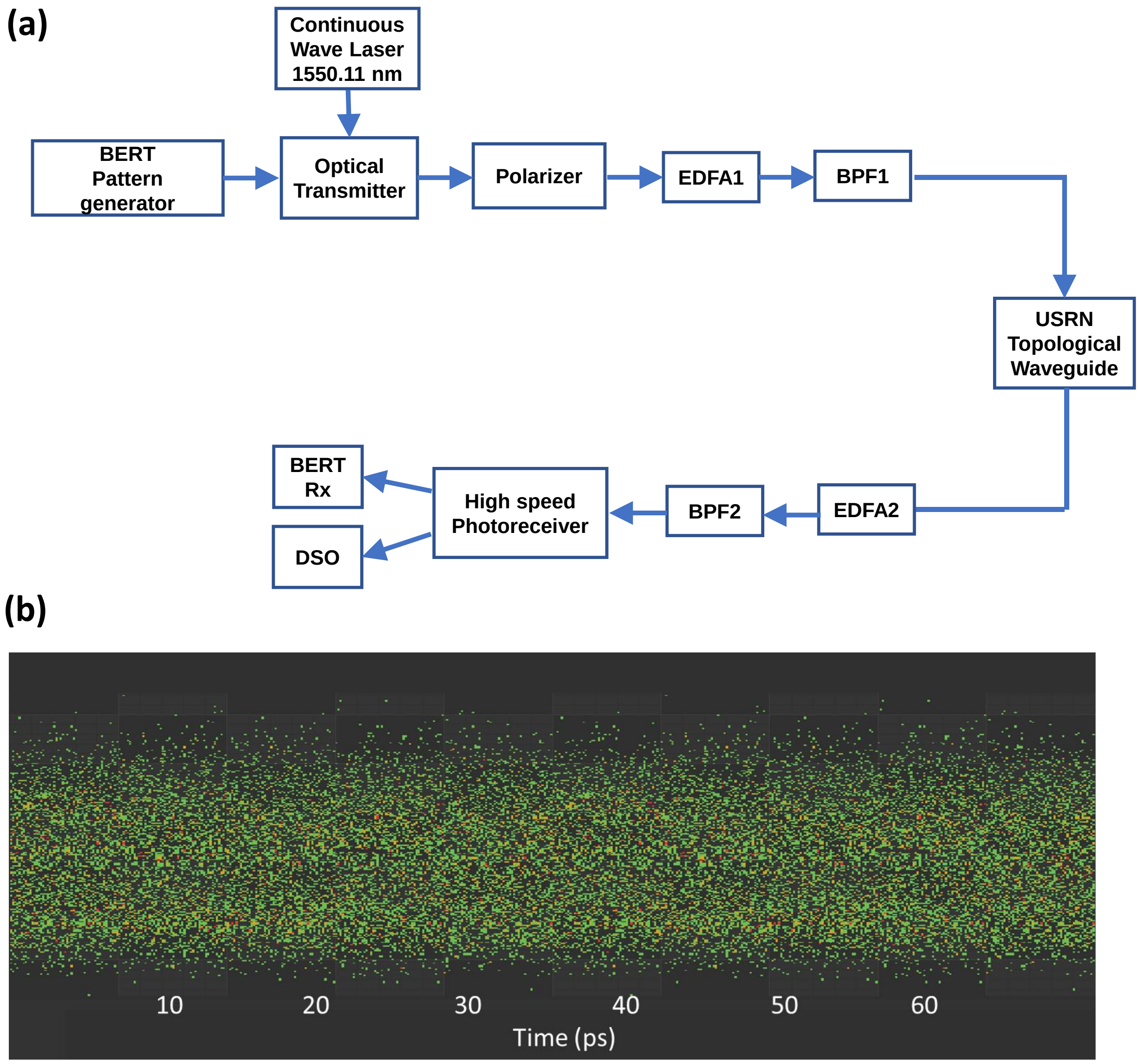


**Supplementary Figure 8 a**, The high-speed test setup used to characterize the transmission of 30 Gb/s NRZ and 56 Gb/s PAM4 data through the USRN topological photonic waveguide. (b) Eye diagram measured at the output of the trivial waveguide array using 30 Gb/s NRZ data.

same as NRZ but using a BERT with PAM4 capability. The characterization is performed against a Back-To-Back (B2B) setup. The B2B setup is the same setup as Supplementary Figure 6 but with the fabricated samples replaced by an optical attenuator whereby its loss is equivalent to the insertion loss of the fabricated sample. The high-speed characterization

results showcase the good linear transmission properties of the USRN topological photonic waveguide and demonstrates their suitability for high-speed data transmission for both NRZ and PAM4 signaling formats, importantly not being bandwidth limited.

We further perform high-speed measurements using a trivial waveguide array where $G_w = G_v = 0.25$ µm, When the signaling format was set to 30 Gb/s NRZ, the eye diagram shown in Supplementary Figure 8 (b) was obtained, where the eye is observed to be close and highly noisy. Varying EDFA1 and EDFA2 to their maximum possible settings did not enable a better eye diagram to be obtained. The corresponding bit error rate could not be obtained because a pattern lock could not be obtained on the BERT Rx. This means that the decoded binary sequence is illegible. This is because the BERT Rx requires a minimum threshold of BER $\leq 10^{-2}$ for the PRBS sequence to be legible. Furthermore, when the signaling format was set to 56 Gb/s PAM4, neither the DSO nor the BERT Rx could achieve a pattern lock. The SSH array is therefore superior to the trivial array in the transmission of high-speed data.

## VII. Supplementary Note 7: Calculation of the signal gain and idler conversion efficiency:

The peak power of the amplified signal may be extracted as $P_{\text{signal,peak}} = \frac{1}{R_{\text{p}}.T} \int P_{\text{signal,ave}}(\lambda) d\lambda$ , where $R_p$ is the repetition rate of the pulses (20 MHz) and $T$ is the pulse width. The peak power of the generated idler is calculated using the expression, $P_{idler,peak} = \frac{1}{R_{\text{p}}.T} \int P_{idler,ave}(\lambda) d\lambda$. $P_{\text{signal,ave}}(\lambda)$ and $P_{\text{idler,ave}}(\lambda)$ denote the measured signal and idler average output power spectrum as a function of wavelength, $\lambda$ [3]. The coupling loss (7 dB) at the output waveguide-fiber interface is added to obtain the idler power out

of the waveguide. The signal gain, $\mathrm{C.E_{signal}} = \frac{P_{\mathrm{signal,peak}}}{P_{\mathrm{signal,out}}}$, where $P_{\mathrm{signal,out}}$ is the signal power at the topological waveguide output with the pump off. The idler conversion efficiency is calculated using, $\mathrm{C.E_{idler}} = \frac{P_{\mathrm{idler,peak}}}{P_{\mathrm{signal,out}}}$.

The theoretical on/off idler conversion efficiency is calculated using the expression, $\mathrm{C.E_{idler}} = [\frac{\gamma P_{\mathrm{in}}}{g} \times \sinh(gL_{\mathrm{eff}})]^2 \cdot \mathrm{e}^{\alpha \cdot L_{\mathrm{eff}}}$, where $P_{\mathrm{in}}$ is the input peak power and the gain coefficient, $g = \left[(\gamma P_{\mathrm{in}})^2 - (\frac{\beta_2 \Omega^2 + 2\gamma P_{\mathrm{in}}}{2})^2\right]^{\frac{1}{2}}$. $L_{\mathrm{eff}}$ is the effective length defined as $\frac{1}{\alpha}[1 - \mathrm{e}^{-\alpha . L}]$, where $\alpha$ and $L$ are the loss coefficient and device length respectively. The nonlinear parameter, $\gamma = \frac{2\pi n_2}{\lambda . A_{\mathrm{eff}}}$ where $n_2$ is the nonlinear refractive index, $\lambda$ is the wavelength and $A_{\mathrm{eff}}$ is the effective area. $\beta_2$ is the group velocity dispersion and $\Omega$ is the detuning between pump and signal. The theoretical on/off signal gain is calculated using the expression, $G_{\mathrm{signal}} = (1 + [\frac{\gamma P_{\mathrm{in}}}{g} \times \sinh(gL_{\mathrm{eff}})]^2) \cdot \mathrm{e}^{\alpha \cdot L_{\mathrm{eff}}}$.

## VIII. Supplementary Note 8: Analysis of thermal effects

While high pump power can induce a localized increase in the refractive index, it may also give rise to thermal effects. Thermal nonlinearities, if applicable, are usually larger than the nonlinearity induced by bound electrons associated with the Kerr effect. The thermal nonlinear effect is most pronounced if the time scale of absorption is less than the order of the temporal pulse width. In USRN, the linear absorption dominates over nonlinear absorption (three-photon absorption) for peak powers of tens of watts [2]. As the pulse energy absorbed by the material is larger for temporally longer pulses, the 1ps laser imposes a

smaller thermal nonlinearity than 5 ps pulses. Thus, the thermal nonlinearity induced by the 1ps pulses may be assumed to be negligible compared to that for 5 ps pulses. If present, the heat generated by the previous pulse could affect subsequent pulses if heat does not dissipate sufficiently before the arrival of the next pulse. The dissipation time should be considered relative to the pulse period. In our sample the dissipation time, $\tau_{\mathrm{D}} = \frac{A_{\mathrm{eff}}}{\alpha} \sim 28.4$ ns, where $\alpha$ is the thermal diffusivity. Since $\tau_{\mathrm{D}} < \frac{1}{R_{\mathrm{P}}} = 50$ ns, thermal effects induced by an optical pulse will dissipate before the arrival of the next pulse. Therefore, effects from thermal nonlinearities are negligible.

## IX. Supplementary Note 9: Zak phase in the SSH system

The bulk topological invariant is defined as the Zak phase, $\gamma = \oint dk \langle \mathbf{u}_k | i \frac{\partial}{\partial k} | \mathbf{u}_k \rangle$, where $k$ is the Bloch wave number and $\mathbf{u}_k$ represents the Bloch states in the Brillouin zone. For a more generalized investigation, we consider couplings between near neighbor waveguides ($v$, $w$), second neighboring waveguides ( $\delta$) and up to the third neighboring waveguides ($\xi$). The coupling coefficients between neighboring waveguides in inter bands are denoted by $v$, while that between neighboring waveguides in intra bands is denoted by $w$. The second neighbor coupling is very weak in our designed system. In our system, the value of coupling coefficient between the second neighbor (two from the defect) is $2.5 \times 10^{-4}$ $\mu m^{-1}$, three orders of magnitude smaller than nearest neighbor couplings. If any, the second neighbor coupling term will merely undergo a shift in eigenvalues. It would have not an effect on the Zak phase. For the third neighboring waveguide coupling terms, the values are very small (less than $10^{-7}$) in our designed system. The Hamiltonian is:

$$H(k) = \int_0^{2\pi} \frac{dk}{2\pi} \begin{pmatrix} A_\mathrm{k}^\dagger & B_\mathrm{k}^\dagger \end{pmatrix} \begin{pmatrix} 2\delta \cos k & v + we^{-ik} + \xi e^{-2ik} \\ v + we^{ik} + \xi e^{2ik} & 2\delta \cos k \end{pmatrix} \begin{pmatrix} A_\mathrm{k} \\ B_\mathrm{k} \end{pmatrix} \tag{13}$$

The corresponding eigenvalues are $2\delta \cos k \pm \sqrt{(v + w\cos k + \xi \cos 2k)^2 + (w \sin k + \xi \sin 2k)^2}$ and eigen vectors $\psi_\pm = \frac{1}{\sqrt{2}}\begin{pmatrix} 1 \\ \pm \mathrm{e}^{\pm i\phi} \end{pmatrix}$, where $\phi = \tan^{-1}((w \sin k + \xi \sin 2k) / (v + w \cos k + \xi \cos 2k))$, the Zak phase is ∓2π, $\mp\pi$ and 0 according to the relationship between coupling coefficients. Supplementary Figure 9 (a) below shows the regional Zak phase distribution for diverse coupling coefficients.

The Zak phase can be calculated from the continuous differential equation. We solved numerically the Bloch solutions for differential equations represented by $u_k(y)$ as $i\frac{\partial \mathbf{u}_k}{\partial z} + \nabla_\perp^2 \mathbf{u}_k - 2ik\frac{\partial}{\partial y}\mathbf{u}_k - k^2\mathbf{u}_k + k_0^2 n_0(y)^2 \mathbf{u}_k = 0$. The eigen values and eigen states are calculated for each $k$ point with 100 points between $-\frac{\pi}{a}$ and $\frac{\pi}{a}$. The Zak phase is calculated using $\gamma = -\sum_{\mathrm{k}_i} \mathrm{Im}[\log\langle \mathbf{u}_{\mathrm{k}_i} | \mathbf{u}_{\mathrm{k}_{i+1}} \rangle]$, as defined in Ref. 6. Supplementary Figure 9 (b) and (c) show that we have two bands, with Zak phase = $\mp\pi$ for $w > v$ ($G_\mathrm{v}$ = 0.25 μm, $G_\mathrm{w}$ = 0.15μm,) and 0 for $v > w$ ($G_\mathrm{v}$ = 0.15 μm, $G_\mathrm{w}$ = 0.25 μm).

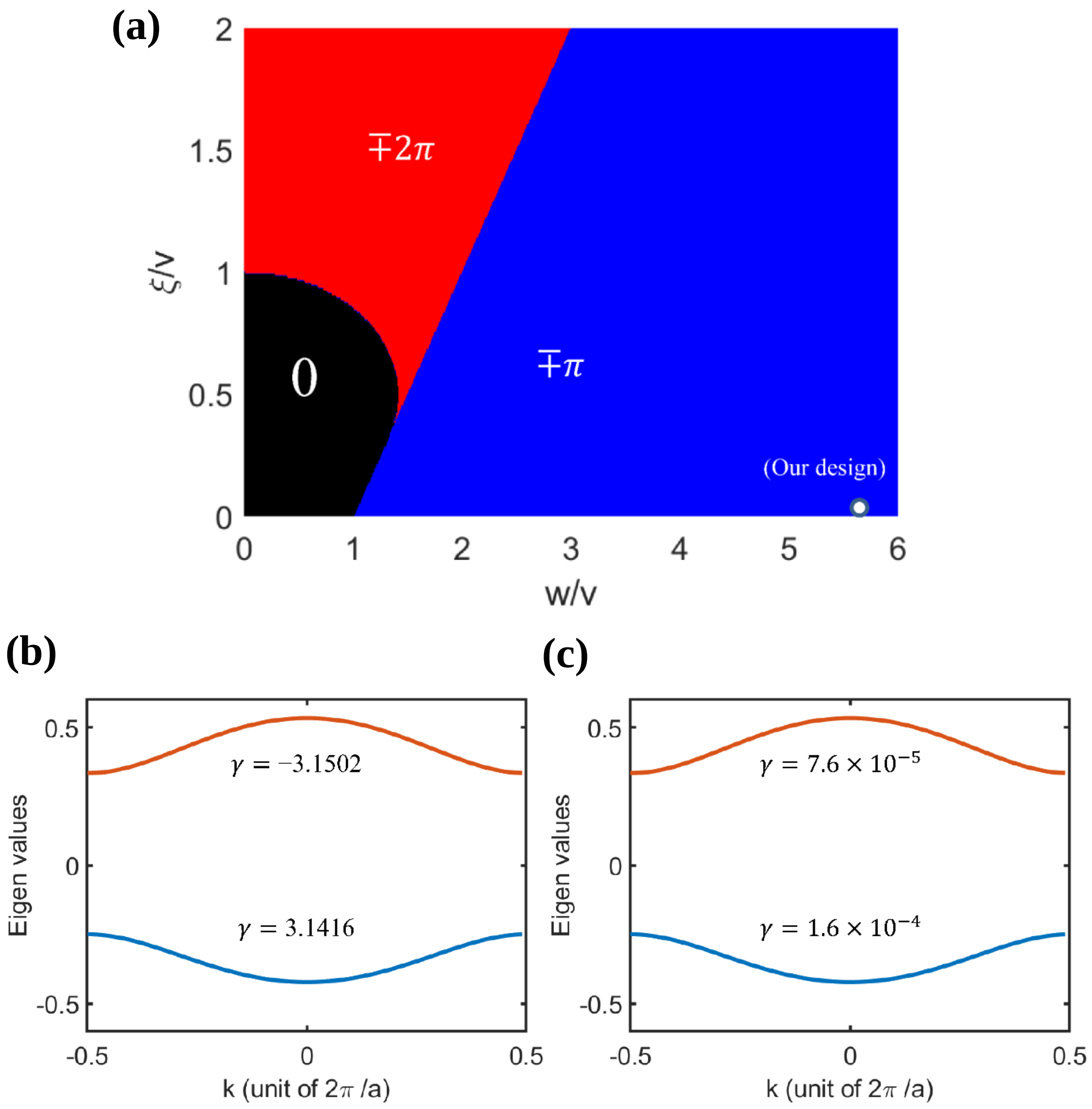


**Supplementary Figure 9 a**, The Zak phase distribution for diverse coupling coefficients, *w*, *v* and $\xi$. The calculated Zak phase in two bands for **b**, $G_v$ = 0.25μm, $G_w$ = 0.15μm (our design) and **c**, $G_v$ = 0.15μm, $G_w$ = 0.25μm.

## X. Supplementary Note 10: 2D effective nonlinear Schrödinger equation for describing the 3D SSH coupled waveguide system

The topological distribution of a refractive index is described by a 3D system. Our SSH array however involves confinement in the vertical direction and does not possess a specific

topological distribution. Thus, the calculation may be simplified by solving a 2D problem with a refractive index distribution as shown in Supplementary Figure 10.

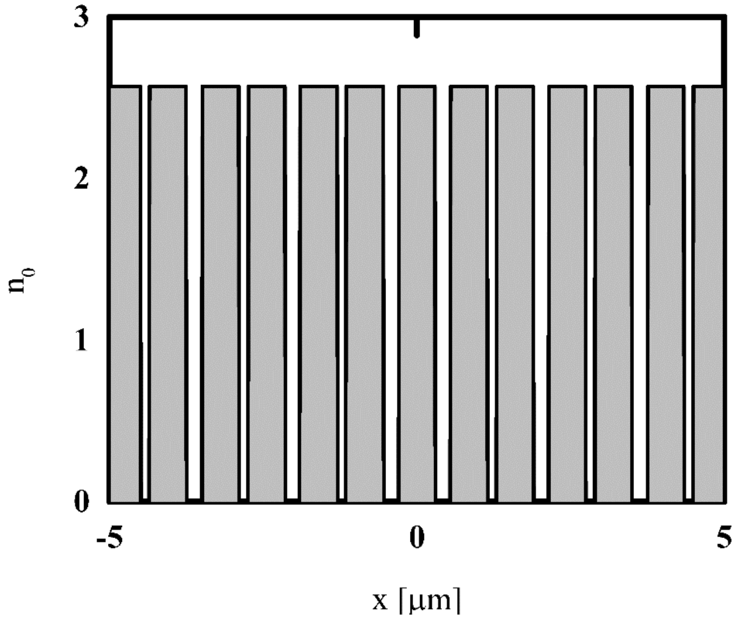


**Supplementary Figure 10** Effective refractive index function for solving the nonlinear Schrödinger equation.

The calculated effective index of this system with reduced dimension is 2.382, whereas the exact effective index calculated using 3D FDTD is 2.386. The boundary modes are almost identical for the 2D and 3D case, as shown in Supplementary Figure 11. Consequently, the system with the reduced dimension provides very similar results as the 3D system.

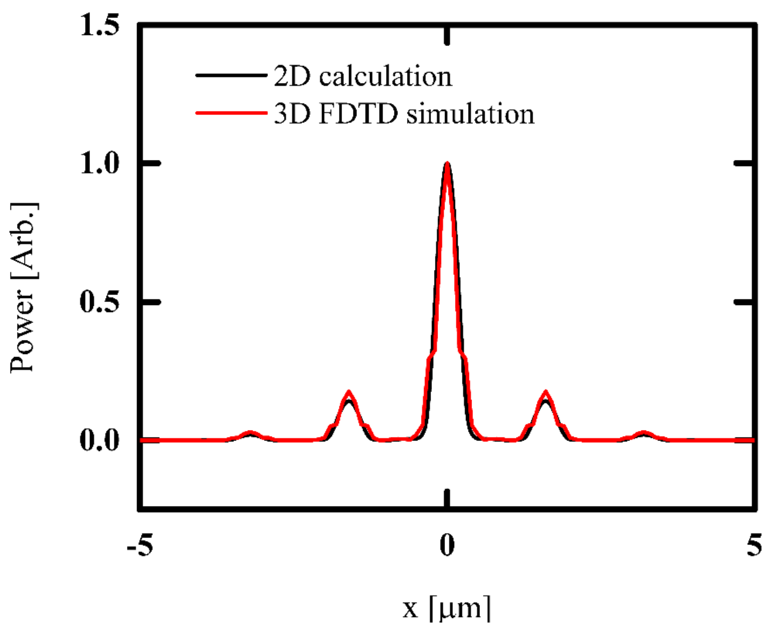


**Supplementary Figure 11** Boundary modes calculated using the 2D nonlinear Schrödinger equation (black) and 3D FDTD (red).

With the effective 2D nonlinear Schrödinger equation, Eigen values and Eigen states satisfying the boundary condition of vanishing fields at $x \to \pm\infty$ are solved as shown in Supplementary Figure 12 (a) – (d). Design parameters including the long/short gap size are studied and observed to enable tuning of the boundary states (Supplementary Figure 12 (d)). When the long gap size is fixed at 0.25 μm, increasing the short gap size from 0.15 μm to 0.25 μm is observed to result in increasing delocalization. Similarly, the nonlinear Kerr effect can also tune the boundary states. The boundary modes are calculated for three different values of $\Delta n$ (the nonlinear refractive index change is further defined by $\Delta n = \frac{3}{4}\epsilon_0 \boldsymbol{\chi}^{(3)}|\mathbf{E}_0|^2$, where $E_0$ is input peak amplitude), with increasing delocalization observed as $\Delta n$ is increased (Supplementary Figure 12 (b)).

Propagation of the optical fields is calculated by solving the 2D nonlinear Schrödinger equation (Supplementary Figure 12 (e) – (g)), where the electric field of the single mode for a single waveguide with same width and thickness as one of waveguides in SSH design is used as the input. For an input field with weak power, the input field is observed to be localized well in the boundary waveguide (Supplementary Figure 12 (e)). As the peak power of the input increases, it is observed that the propagation is no longer well localized in the boundary waveguide. It is observed from Supplementary Figure 12 (f) where $\Delta n = 0.013$, propagation localized to the boundary waveguide no longer holds as a result of the eigen value of the boundary state crossing from the forbidden bandgap region to the bulk states region. Lastly, propagation in the trivial regime is shown in Supplementary Figure 12 (g) where it is observed that the light propagation spreads out akin to spatial diffraction along the propagation direction, similar to that observed in Supplementary Figure 6 (a).

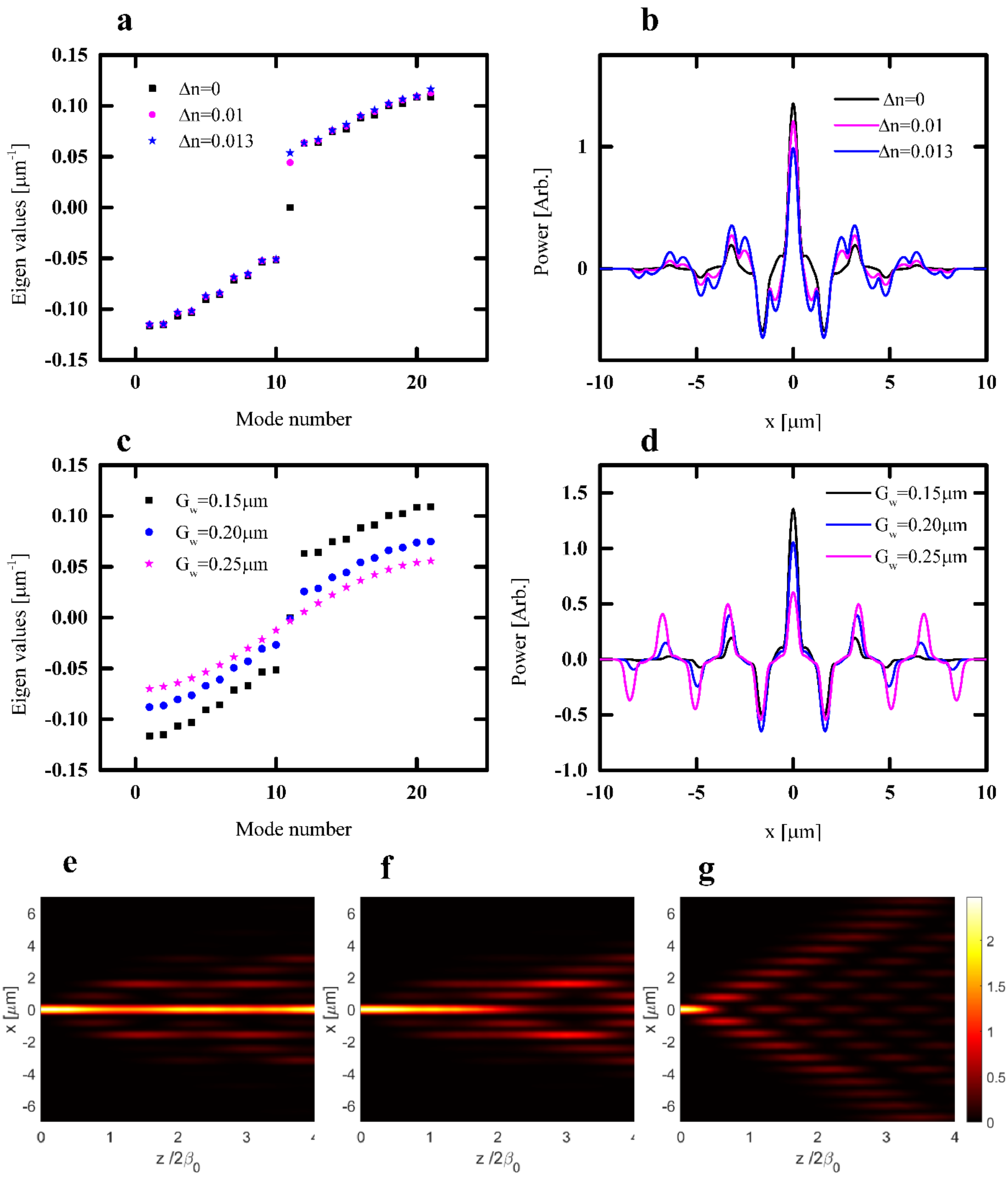


**Supplementary Figure 12** Theoretical calculations using the nonlinear Schrödinger equation for **a**, Eigen energy bands and **b**, eigenstates as a function of refractive index changes induced by the nonlinear Kerr effect. **c**, Eigen energy bands and **d**, eigenstates as a function of various short gap sizes. The propagation of the fields for **e**, weak power in the SSH regime, **f**, strong power in the SSH regime, and **g**, in the trivial regime.

## XI. Supplementary Note 11: Discretized Hamiltonian from the nonlinear differential equations

The nonlinear Schrödinger equation is represented by a Hamiltonian matrix with basis of single modes in single waveguides with different locations. $K_{i,j} = \langle \mathbf{TE}_{00,i} \left| \frac{1}{2\,\beta_0} \boldsymbol{\nabla}_\perp^2 + \frac{k_0^2 n_0^2 - \beta_0^2 + 2\,k_0^2 n_0 n_2 |\mathbf{E}|^2}{2\beta_0} \right| \mathbf{TE}_{00,j} \rangle$ and $\mathbf{E} = \sum_i A_i |\mathbf{TE}_{00,i}\rangle$, where $i$ represents the index of the waveguides, $|\mathbf{TE}_{00}\rangle$ is a TE single mode and $\mathrm{A}_i$ is amplitude of fields for the ith waveguide. The nonlinear Schrödinger equation is represented using a matrix equation, $i\frac{\partial A_i}{\partial z} = \widehat{\mathbf{K}_0}_{ij} A_j - \frac{k_0^2}{\beta_0} n_0 n_2 |A_i|^2 \delta_{i,j} A_j$, where $\widehat{\mathbf{K}_0} = -\frac{1}{2\,\beta_0}\boldsymbol{\nabla}_\perp^2 - \frac{k_0^2 n_0^2 - \beta_0^2}{2\beta_0}$ is a linear operator and $\frac{k_0^2}{\beta_0} n_0 n_2 |A_i|^2 \delta_{i,j} A_j$ is nonlinear operator with only diagonal terms. $K_{ii}$=0 when $\beta_0 = n_{\mathrm{eff}} k_0$, where $n_{\mathrm{eff}}$ is the effective refractive index for a boundary mode. The off-diagonal terms, $K_{0,ij} = -\frac{k_0^2}{2\beta_0} \langle \mathbf{TE}_{00,i} | n_0^2 | \mathbf{TE}_{00,j\neq i} \rangle$ are usually referred to as the coupling coefficient.

This approach reduces the computational time and power required to solve the nonlinear Schrödinger equation, especially since the implemented system consists of a large number of waveguides (199). To verify the accuracy of the discretized Hamiltonian method compared to calculations using the nonlinear Schrödinger equation, we calculate the boundary mode using both methods as shown in Supplementary Figure 13. It is observed that both methods give similar results.

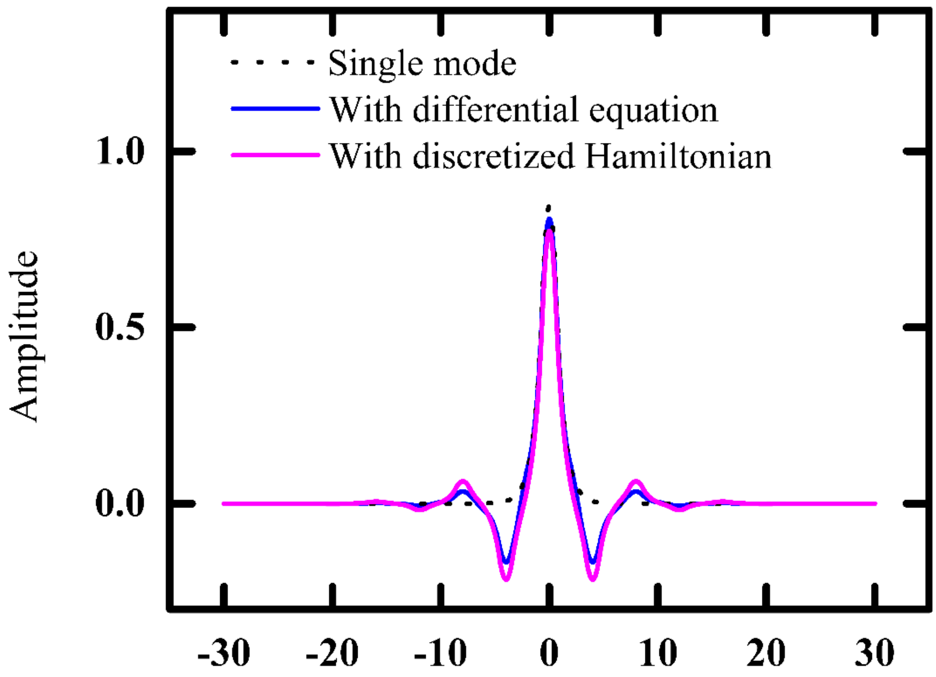


**Supplementary Figure 13** Defect mode distribution calculated by solving the differential equation (blue line) and discretized Hamiltonian (magenta line). The black dotted line corresponds to a single mode for a single waveguide.

With the discretized Hamiltonian, the transmittance may be solved at a specific waveguide of output according to,

$$T_{center} = \left( |\langle \mathbf{0}|\boldsymbol{\psi}_0\rangle|^2 + 2\sum_{\kappa_i>0}|\langle \mathbf{0}|\boldsymbol{\psi}_i\rangle|^2 \cos\kappa_i z \right)^2, \quad (14)$$

where $|\boldsymbol{\psi}_i\rangle, |\boldsymbol{\psi}_0\rangle$ represents the eigenstates for the bulk and boundary respectively, *i* is the eigenstate index, $\kappa_i$ are eigenvalues and $|i\rangle$ is a single mode for a single waveguide having waveguide index *i*. The first term is fixed and describes propagation of the zero mode while the second term describes a modulation along the propagation direction. The scale of modulation is given by $2\langle \mathbf{0}|\boldsymbol{\psi}_i\rangle|^2$. These values are not small (~0.31) despite the tight localization of the topological mode. However, they decay to zero as propagation

progresses because of very strong dephasing between other bulk states as illustrated in Supplementary Figure 14. Similarly, the transmittance of the nearest neighbor can be calculated as follows:

$$T_{\text{near}} = \langle \mathbf{1}|\boldsymbol{\psi}_0\rangle\langle\boldsymbol{\psi}_0|\mathbf{0}\rangle + \sum_i \langle \mathbf{1}|\boldsymbol{\psi}_i\rangle\langle\boldsymbol{\psi}_i|\mathbf{0}\rangle\, e^{-i\kappa_i z} \quad (15)$$

The transmittance for the 1st and 2nd neighboring waveguides is further plotted in Supplementary Figure 14.

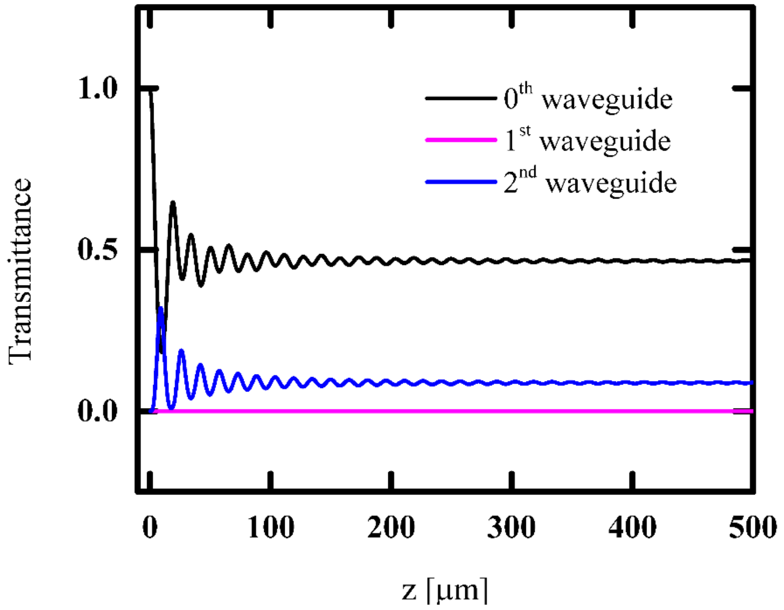


**Supplementary Figure 14** Modulated transmittance at the output of 0th, 1st, and 2nd waveguides in the SSH system when a non-defect mode is input into the system (eg. A single mode of a single waveguide).

## XII. Supplementary Note 12: Optical parametric gain performance comparison between SSH and trivial arrays

We investigate the efficiency of the parametric process when occurring in the SSH array vs. a trivial array. The pump (5 ps pulses) and signal (continuous-wave) are coupled into the topological and trivial samples and the output from the center waveguide is measured

using an OSA. Parametric gain or conversion could not be observed in the trivial array as shown in Supplementary Figure 15 (a). Poor localization of the light to the center waveguide, also predicted through experiments (Supplementary Figure 7) and simulations (Supplementary Figure 12(g)) would lead to low conversion efficiencies.

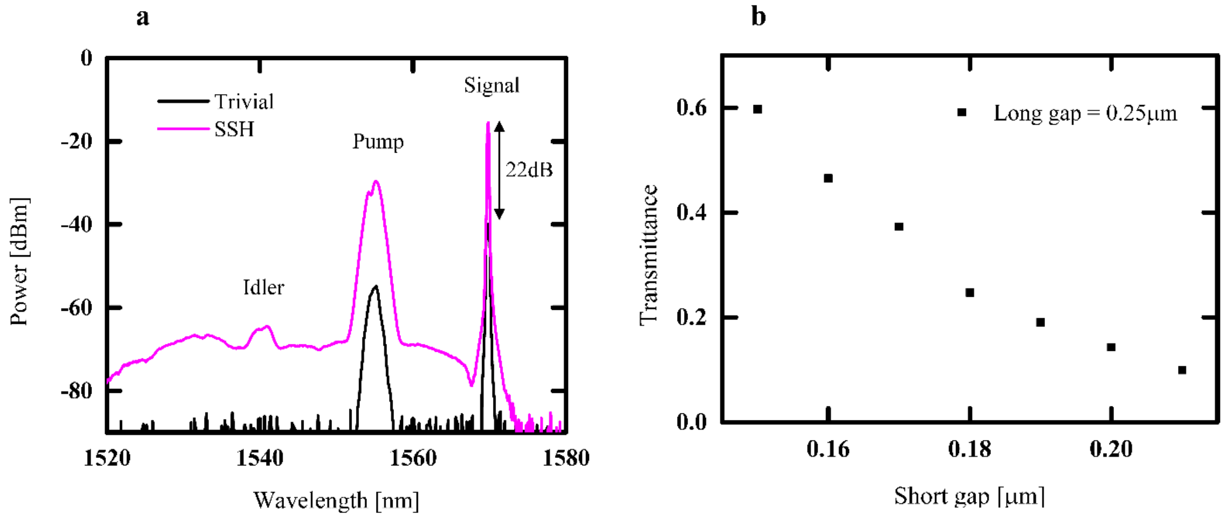


**Supplementary Figure 15 a**, Four wave mixing measurement at a trivial sample with same gap distance (black line) and a SSH designed sample (magenta line) **b**, Calculated transmittance versus short gap distance ($G_w$) for a fixed long gap distance ($G_v$ = 0.25 μm).

## XIII. Supplementary Note 13: Theoretical calculations for the four-wave mixing process

We calculate the propagation dynamics for the co-propagating pump, signal, and generated idler fields using the coupled nonlinear Schrödinger equation as follows,

$$i2\beta_{0p}\frac{\partial \mathbf{E}_p}{\partial z} + \nabla_\perp^2 \mathbf{E}_p + (k_{0p}^2 n_0^2 - \beta_{0p}^2)\mathbf{E}_p + 2k_{0p}^2 n_0 \mathbf{n}_2 \left(\left|\mathbf{E}_p\right|^2 + 2|\mathbf{E}_s|^2 + 2|\mathbf{E}_i|^2\right)\mathbf{E}_p +$$

$$4k_{0p}^2 n_0 \mathbf{n}_2 \mathbf{E}_p^* \mathbf{E}_s \mathbf{E}_i e^{i\Delta\beta z} = 0 \qquad (16a)$$

$$i2\beta_{0s}\frac{\partial \boldsymbol{E}_s}{\partial z} + \nabla_\perp^2 \mathbf{E}_s + (k_{0s}^2 n_0^2 - \beta_{0s}^2)\mathbf{E}_s + 2k_{0s}^2 n_0 \mathbf{n}_2 \left(|\mathbf{E}_s|^2 + 2|\mathbf{E}_p|^2 + 2|\mathbf{E}_i|^2\right)\mathbf{E}_s + 4k_{0s}^2 n_0 \mathbf{n}_2 \mathbf{E}_s^* \mathbf{E}_p \mathbf{E}_i e^{-i\Delta\beta z} = 0 \quad (16b)$$

$$i2\beta_{0i}\frac{\partial \boldsymbol{E}_i}{\partial z} + \nabla_\perp^2 \mathbf{E}_i + (k_{0i}^2 n_0^2 - \beta_{0i}^2)\mathbf{E}_i + 2k_{0i}^2 n_0 \mathbf{n}_2 \left(|\mathbf{E}_i|^2 + 2|\mathbf{E}_p|^2 + 2|\mathbf{E}_s|^2\right)\mathbf{E}_i + 4k_{0i}^2 n_0 \mathbf{n}_2 \mathbf{E}_i^* \mathbf{E}_p \mathbf{E}_s e^{-i\Delta\beta z} = 0 \quad (16c)$$

where $\Delta\beta = \beta_{0i} + \beta_{0s} - 2\beta_{0p}$.

The propagation of the input (a) pump, (b) signal and (c) generated by four-wave mixing is calculated as shown in Supplementary Figure 16. The calculation is performed over a shorter length (~80 μm) that is shorter than our sample's length (4 mm) but exceeds one period of the modulation length associated with SSH structure. Thus, the calculated gain for the full 4 mm device length will be 33 dB larger since the generated power is proportional to the square of the phase matched length.

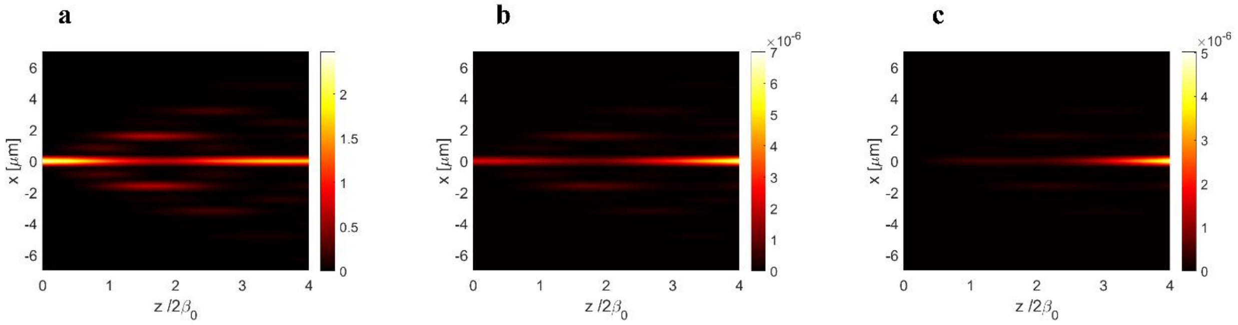


**Supplementary Figure 16** Calculated fields propagation for **a**, a pump beam, **b**, a signal beam and **c**, a generated idler beam in optical parametric amplification dynamics in a SSH system.

Our calculations shown in Supplementary Figure 17 also reveal that the optical parametric gain saturates at high peak power. The observed saturation occurs when $\Delta n \sim 0.0065$, before the boundary state crosses the band gap where transmission saturation occurs ($\Delta n = 0.013$). Transmission saturation in turn occurs because of the two times stronger pump-signal coupling compared to the extent of self-phase modulation.

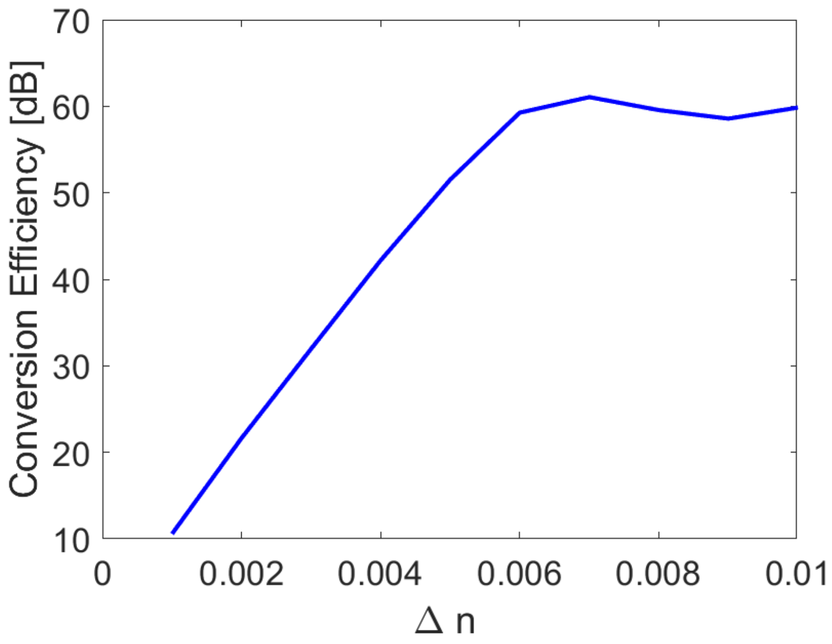


**Supplementary Figure 17** Saturation of optical parametric gain at high input peak power.

In addition, we perform calculations using the coupled equations to study the impact of short gap size on the signal gain and idler conversion efficiency. The long gap size is fixed at 0.25 μm and we use $\Delta n = 0.0013$, corresponding to the value in our experiment. It is observed from Supplementary Figure 18 that the signal gain and conversion efficiency decrease as the short gap size approaches the long gap size of 0.25 μm. The signal gain approaches a value of zero. The reduced conversion efficiency and signal gain observed arise from poor localization of the boundary state.

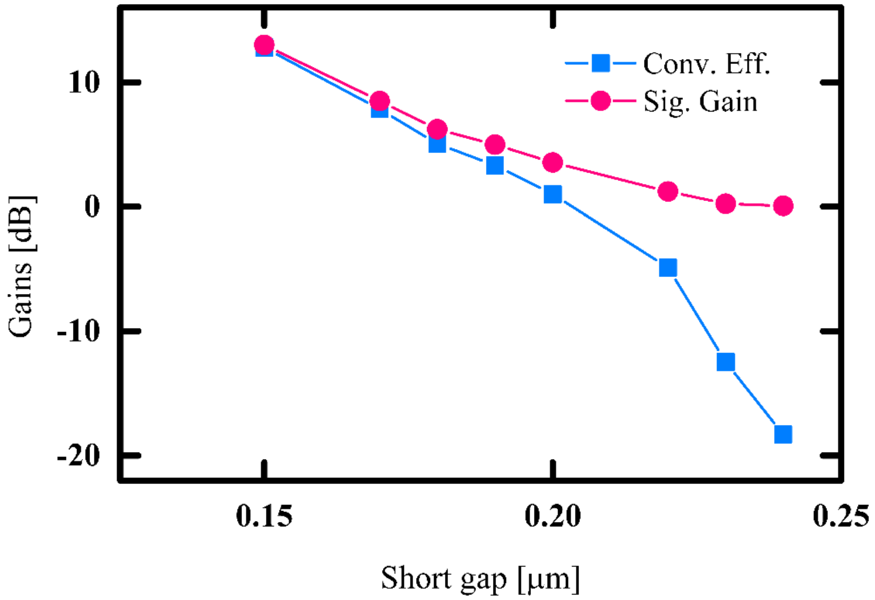


**Supplementary Figure 18** Calculated signal gain and conversion efficiency as a function of short gap size for a fixed long gap size of 0.25 μm. The calculation is performed using the coupled equations, for $\Delta n$ = 0.0013, corresponding to the value in our experiments.

## XIV. Supplementary Note 14: Discussion on advantages of the topological system over a single waveguide

The topological waveguide offers an advantage over a single waveguide, in that its linear transmission can be tuned more widely. By varying the power in the central waveguide perhaps as a switching power, the local refractive index changes and offers a knob to change the absolute power transmitted via the extent of modal delocalization.

Another point of interest is that this topological system experiences a variation in the power as it propagates. In a single waveguide, linear transmission properties are such that the power, $P$ at any point of the waveguide varies because of propagation losses ($P = P_0 e^{-\alpha z}$,

$P_0$ is the input power, $\alpha$ is the attenuation coefficient and $z$ is the propagation distance). There is perhaps a possibility to harness the power modulation in the topological system to design devices which provide a variation in the output power based on the length.

If we could input a boundary mode type of beam, the linear transmission would be identical to that of a single waveguide, but the transmission could be implemented by a topological boundary mode with a lesser degree of localization compared to a single waveguide. The less localized wavefunction would reduce nonlinear loss effects such as multi-photon absorption. The less localized wavefunction wave function would also reduce the effective index and provide an additional design knob to tailor dispersion.

In addition, the topological waveguide provides some robustness to disorder that can be introduced from fabrication processes. For example, there could be slight variations in the waveguide positions and coupling coefficients that arise from the lithographic process. Such SSH waveguides have been shown to be robust against disorder in coupling coefficients [7] and waveguide position[8]. This unique feature is an advantage in the transmission provided by the topological waveguide over the single waveguide.

The topological waveguide also provides an additional advantage over a single waveguide when considering the possible sources of loss. There are two contributions to the loss in the topological system: (1) internal loss, described by the loss coefficient, $\alpha$ and (2) topological loss, denoted by $\delta n_0$, as shown in the nonlinear Schrödinger equation below. The internal loss is induced from surface roughness of the structure. Conversely, topological loss originates from other effects which impact the topological mode such as refractive index distribution of waveguides or perturbations in the gap distance which could cause chiral symmetry to be broken. If disorder induced chiral symmetry breaking is

insufficient to close the band gap, the topological boundary state will be maintained as documented in Refs. 7 and 8.

In a single waveguide, loss originates from surface roughness. The roughness could hop a guided mode via scattering to excite higher order modes depending on the extent of roughness. The higher order modes are not a guided mode in our single mode waveguide. The waveguide has two transverse directions. The vertical direction ($x$-axis) and the horizontal direction ($y$-axis) corresponding to the topological distribution in the SSH system. We may further analyze the disorder from roughness in the topological SSH system and how that compares with roughness in a single waveguide.

Roughness increases the linear loss coefficient, $\alpha$. For the topological system, the linear loss is considered in the nonlinear Schrödinger Hamiltonian. The linear loss could be included in a diagonal term. Thus, the linear loss does not impact the integrity of topological states. Nevertheless, the boundary state could be compromised by the introduction of imaginary quantities (linear loss), inducing non-Hermicity in the Hamiltonian. If the randomness of loss coefficients in the waveguide array is not sufficiently large to go over the band gap, we expect that the effect is too small to cause the boundary state to no longer exist. The effect of linear loss may be considered using the nonlinear Schrödinger equation,

$$i\frac{\partial \mathbf{E}}{\partial z} + i\alpha\mathbf{E} + \frac{1}{2\beta_0}\nabla_\perp^2\mathbf{E} + \frac{k_0^2(n_0+\delta n_0)^2 - \beta_0^2 + 2\,k_0^2 n_0 n_2|\mathbf{E}|^2}{2\beta_0}\mathbf{E} = 0$$

For the purpose of providing qualitative insight into the loss mechanism, we utilize the discrete model. The matrix form for the linear loss part can be represented as follows:

$$\alpha_{i,j} = \langle \mathbf{TE}_{00,i} | \alpha | \mathbf{TE}_{00,j} \rangle = \begin{pmatrix} \ddots & 0 & 0 & 0 & 0 \\ 0 & \alpha_1 & 0 & 0 & 0 \\ 0 & 0 & \alpha_0 & 0 & 0 \\ 0 & 0 & 0 & \alpha_{-1} & 0 \\ 0 & 0 & 0 & 0 & \ddots \end{pmatrix}$$

$$= \alpha_{\mathrm{av}} + \begin{pmatrix} \ddots & 0 & 0 & 0 & 0 \\ 0 & \Delta\alpha_1 & 0 & 0 & 0 \\ 0 & 0 & \Delta\alpha_0 & 0 & 0 \\ 0 & 0 & 0 & \Delta\alpha_{-1} & 0 \\ 0 & 0 & 0 & 0 & \ddots \end{pmatrix}$$

The average loss term, $\alpha_{\mathrm{av}} = 1/N \sum_i \alpha_{\mathrm{i}}$ and $\Delta\alpha_{\mathrm{i}} = (\alpha_{\mathrm{i}} - \alpha_0)$. If $\alpha_{\mathrm{av}} \gg \Delta\alpha_i$ is satisfied, the loss terms may be represented by $\alpha_{\mathrm{av}}$ as the dominant term. The term gives the topological boundary mode an imaginary shifted eigen value and exponentially decaying eigen function. Thus, the loss cannot be removed by the SSH topological design. In this case, we may consider scattering in two transverse directions. In the vertical direction (*x*-axis), the scattering to non-confined modes cannot be avoided and the effect may be introduced as a loss coefficient. In the horizontal direction (*y*-axis) however, which possesses a topological distribution dimension, if roughness induces sufficiently large scattering between two neighboring waveguides in the SSH array, an off-diagonal term could be introduced and impact the topological states. However, we expect this quantity to be so small in our system that it would be far from sufficient to close the band gap of bulk states. Consequently, light scattered in the *y*-direction is protected topologically.

Conversely for a single waveguide, light scattered from roughness scatters not only in the *x*-direction but also the *y*-direction. The SSH system on the other hand is topologically protected in the *y*-axis. Thus, roughness induced reductions in transmittance are smaller in the SSH device than in a single waveguide.

## XV. Supplementary Note 15: Validity for slow varying approximation in the tightly focused topological system

The slowly varying envelope approximation was used to derive the paraxial diffraction equation from Maxwell equation. The phase acquired in the propagation direction (pertains to $k_z$) is dominant and fast varying, whereas any $k_x$ and $k_y$ divergent terms owing to the wave property or uncertainty relation will come into the field envelope, $A(x,y,z) \in$ C, and hence the electric-field, $E(x,y,z) = A(x,y,z)e^{i\, n_{\mathrm{eff}} k_z z}$. To satisfy conditions for the slowly varying envelope approximation, any transverse variation of the field envelope should be less than the fast phase variation occurring in the field's propagation direction:

$$\frac{\partial}{\partial x}A(x,y,z)/A(x,y,z)\ or\ \frac{\partial}{\partial y}A(x,y,z)/A(x,y,z)\ or\ \frac{\partial}{\partial z}A(x,y,z)/A(x,y,z) \ll n_{\mathrm{eff}}k_z$$

Phase variations in the *x* and *y* directions would be determined from the field confinement via the uncertainty relation or from the exponentially decaying function describing the *A*(*x,y,z*) fields in the cladding region. $\frac{\frac{\partial}{\partial x}A(x,y,z)}{A(x,y,z)} \sim \frac{\frac{\partial}{\partial y}A(x,y,z)}{A(x,y,z)} \sim 1/\sqrt{a_{\mathrm{eff}}}$ =1.6 $\mu\mathrm{m}^{-1}$ and $\frac{\frac{\partial}{\partial z}A(x,y,z)}{A(x,y,z)} = \pi K$ , where $K$ is the coupling coefficient. In our design, $\pi K = 0.12\ \mu\mathrm{m}^{-1}$ and $n_{\mathrm{eff}}k_0 = \frac{n_{\mathrm{eff}}2\pi}{\lambda} = 9.7\ \mu\mathrm{m}^{-1}$ . Thus, the slowly varying envelope approximation is well satisfied.

## References


1. Sohn, B.-U., Choi, J. W., Ng, D. K. & Tan, D. T. Optical nonlinearities in ultra-silicon-rich nitride characterized using z-scan measurements. Sci. Rep. 9, 1-7 (2019).

2. Wang, T. et al. Supercontinuum generation in bandgap engineered, back-end CMOS compatible silicon rich nitride waveguides. Laser Photonics Rev. 9, 498-506 (2015).

3. Ooi, K. *et al.* Pushing the limits of CMOS optical parametric amplifiers with USRN: Si 7 N 3 above the two-photon absorption edge. *Nat. Commun.* **8**, 1-10 (2017).

4. De Dobbelaere, P. et al. in 2017 IEEE International Electron Devices Meeting (IEDM). 34.31. 31-34.31. 34 (IEEE).

5. Nagarajan, R. et al. Silicon photonics-based 100 Gbit/s, PAM4, DWDM data center interconnects. J. Opt. Commun. Netw. 10, B25-B36 (2018).6 Ooi, K. et al. Pushing the limits of CMOS optical parametric amplifiers with USRN: Si 7 N 3 above the two-photon absorption edge. Nat. Commun. 8, 1-10 (2017).

6. Takahiro, F., Yasuhiro, H. & Hiroshi, S. Chern Numbers in Discretized Brillouin Zone: Efficient Method of Computing (Spin) Hall Conductances. J. Phys. Soc. Jpn. (2013).

7. Blanco-Redondo, A., Bell, B., Oren, D., Eggleton, B. J. & Segev, M. Topological protection of biphoton states. Science 362, 568-571 (2018).

8. Wang, M. et al. Topologically protected entangled photonic states. Nanophotonics 8, 1327-1335 (2019).
